# Synthesis of New Pyrazolo[3,4-*d*]pyrimidine Derivatives: NMR Spectroscopic Characterization, X-Ray, Hirshfeld Surface Analysis, DFT, Molecular Docking, and Antiproliferative Activity Investigations

**DOI:** 10.3390/molecules29215020

**Published:** 2024-10-24

**Authors:** Mohamed El Hafi, El Hassane Anouar, Sanae Lahmidi, Mohammed Boulhaoua, Mohammed Loubidi, Ashwag S. Alanazi, Insaf Filali, Mohamed Hefnawy, Lhoussaine El Ghayati, Joel T. Mague, El Mokhtar Essassi

**Affiliations:** 1 Faculty of Medicine and Pharmacy, Mohammed First University, Oujda 60000, Morocco; elhafi.mohamed1@gmail.com; 2 Laboratory of Heterocyclic Organic Chemistry, Department of Chemistry, Faculty of Sciences, Mohammed V University in Rabat, Rabat 10106, Morocco; lahmidi_sanae@yahoo.fr (S.L.); mboulhaoua@gmail.com (M.B.); lelghayati2@gmail.com (L.E.G.); emessassi@yahoo.fr (E.M.E.); 3 Department of Chemistry, College of Science and Humanities in Al-Kharj, Prince Sattam bin Abdulaziz University, Al-Kharj 11942, Saudi Arabia; insaf_filali@yahoo.fr; 4 Institute of Chemistry, ELTE Eötvös Loránd University, Pázmány P. sétány 1/A, 1117 Budapest, Hungary; 5 Department of Chemistry, Faculty of Sciences Semlalia, Cadi Ayyad University, Marrakech 40000, Morocco; m.loubidi@gmail.com; 6 Department of Pharmaceutical Sciences, College of Pharmacy, Princess Nourah bint Abdulrahman University, Riyadh 11671, Saudi Arabia; asalanzi@pnu.edu.sa; 7 Department of Pharmaceutical Chemistry, College of Pharmacy, King Saud University, Riyadh 11451, Saudi Arabia; mhefnawy@ksu.edu.sa; 8 Department of Chemistry, Tulane University, New Orleans, LA 70118, USA; joelt@tulane.edu

**Keywords:** pyrazolopyrimidines, crystal structure, DFT, molecular docking, antiproliferative activity

## Abstract

Four new pyrazolo[3,4-*d*]pyrimidines (**P1**–**P4**) were successfully synthesized in good relative yields by reacting 3-methyl-1-phenyl-1H-pyrazolo[3,4-*d*]pyrimidin-4-ol with various alkylating agents (methyl iodide, propargyl bromide, and phenacyl bromide) at room temperature in DMF solvent, employing liquid–solid phase transfer catalysis. The **P1**–**P4** structures were elucidated using NMR spectroscopy and X-ray diffraction. Intermolecular interactions in **P1**–**P4** were analyzed via Hirshfeld surface analysis and 2D fingerprint plots. Geometrical parameters were accurately modeled by DFT calculations using the B3LYP hybrid functional combined with a 6–311++G(d,p) basis set. The antiproliferative activity of **P1**–**P4** towards colorectal carcinoma (HCT 116), human hepatocellular carcinoma (HepG2), and human breast cancer (MCF-7) cell lines, along with one normal cell line (WI38) was investigated using the MTT assay and sunitinib as a reference. Compounds **P1** and **P2** exhibited antiproliferative activities comparable to the reference drug towards all tested cells, with an IC_50_ range of 22.7–40.75 µM. Both compounds also showed high selectivity indices and minimal cytotoxic effects on the normal cell line. Molecular docking revealed that the significant antiproliferative activity may attributed to the number and type of intermolecular hydrogen bonding established between pyrazolo[3,4-*d*]pyrimidines and DNA topoisomerase, a common target for various anticancer agents.

## 1. Introduction

Cancer is one of the foremost causes of the increase in the number of deaths and morbidity around the globe, mainly in the aging population, and appears to be more likely related to changes in unhealthy lifestyles. Various cancers are categorized according to the damaged tissue or organ. Certain cancers have well-defined mechanisms through which normal cells become cancerous. In the last decades of the 20th century, oncological studies reported that most cancerous cells exhibit a few acquired molecular, biochemical, and cellular characteristics that result from the alteration of key pathways [1]. Heterocycles are the main component of the widely available anti-cancer drugs; the FDA reported that two-thirds of anti-cancer agents contain heterocycle rings. In recent years, there has been increased interest in the chemistry of pyrazolo[3,4-*d*]pyrimidines owing to their wide-ranging biological activities, such as anticancer [2], antibacterial [3], antitubercular [4], and antitumor effects [5].

**Figure 1:**
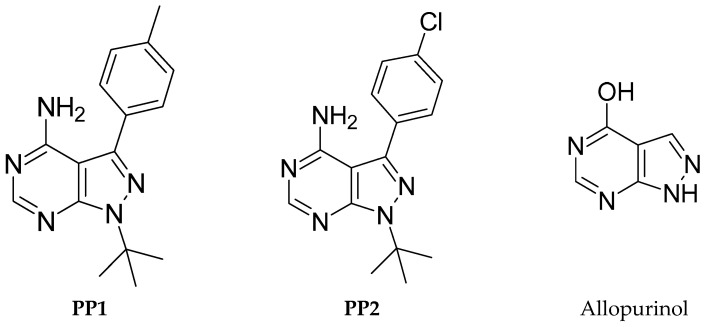
Chemical structures of reported pyrazolo[3,4-*d*]pyrimidine derivatives.

This nucleus constitutes a class of heterocyclic compounds of interest both chemically and pharmaceutically, as they were found to exhibit their antitumor activity by inhibiting different types of enzymes such as Bruton’s tyrosine kinase (BTK) [6], Src tyrosine kinase [7,8], Myt1 kinase [9], and TRAP1 kinase [10]. To the best of our knowledge, the first pyrazolo[3,4-*d*]pyrimidine kinase inhibitors identified were 4-amino-5-(4-methylphenyl)-7-(t-butyl)pyrazolo[3,4-*d*]pyrimidine (**PP1**) and 4-amino-5-(4-chlorophenyl)-7-(t-butyl)pyrazolo[3,4-*d*]pyrimidine (**PP2**) (Figure 1), which were first discovered to act as kinase inhibitors of the SRC family of non-receptor tyrosine kinases in 1996 [11]. Indeed, pyrazolo[3,4-*d*]pyrimidines have several active sites, giving them a high reactivity, making these heterocycles excellent precursors in synthesizing new heterocyclic systems likely to present a broad spectrum of activity. For example, Allopurinol (Figure 1), first synthesized by Robins is used in human clinics as a drug in the treatment of gout, a condition characterized by increased levels of uric acid in the blood [12]. Accordingly, synthesizing new pyrazolo[3,4-*d*]pyrimidines has attracted more attention using various synthetic methods [13,14,15,16,17].

Given the importance that pyrazolo[3,4-*d*]pyrimidine derivatives brought to the biological and therapeutic fields and in continuation of our previous studies [18,19], we report the synthesis and structural elucidation of four new pyrazolo[3,4-*d*]pyrimidines (**P1**–**P4**) that have diverse groups at position 1 of the pyrazolopyrimidine core. Their structures were elucidated by X-ray crystallography and NMR spectroscopy. Additional studies of the new molecules included Hirshfeld surface analysis, DFT calculations, molecular docking, and in vitro antiproliferative investigations.

## 2. Results and Discussion

### 2.1. X-Ray Structure Descriptions

#### 2.1.1. Crystal Structure of 3-methyl-1-phenyl-1,5-dihydro-4H-pyrazolo[3,4-*d*]pyrimidin-4-one (**P1**)

**Figure 2:**
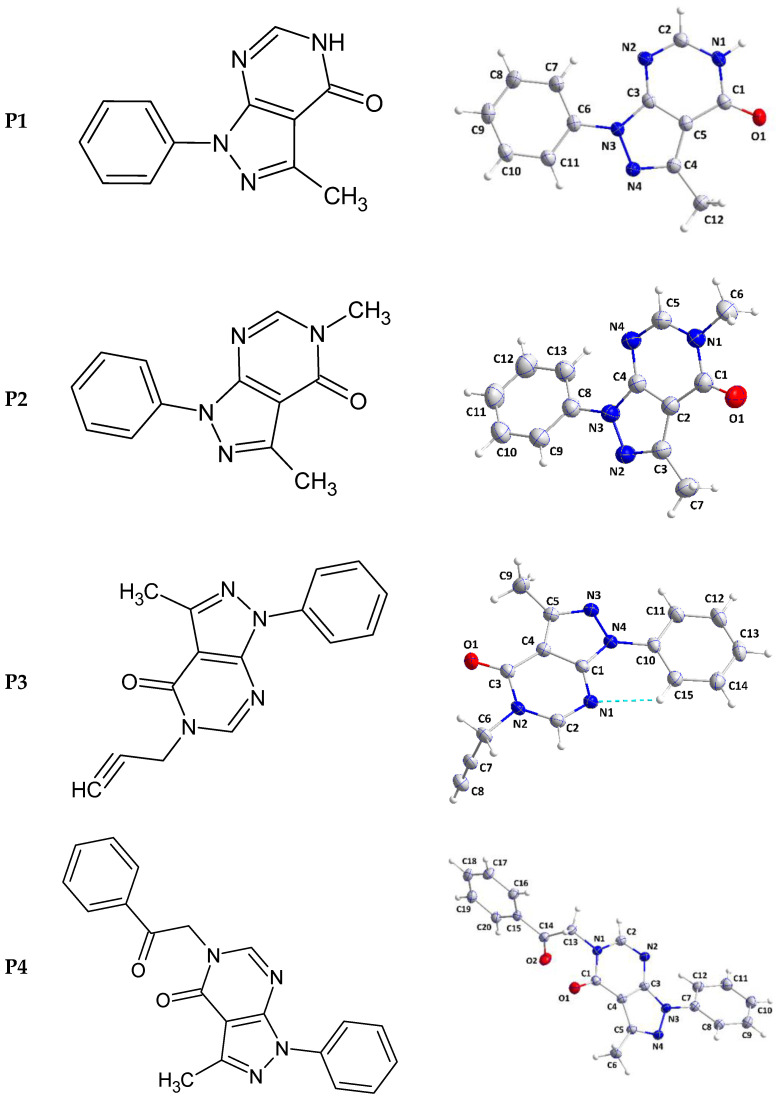
2D structures (**left**) and perspective views of **P1**–**P4** (**right**). The dashed line in **P3** represents the intramolecular hydrogen bond.

The pyrazolopyrimidine unit is slightly non-planar as indicated by the dihedral angle of 1.22(8)° between the mean planes of the constituent rings. The mean plane of the C6···C11 benzene ring is inclined to that of the pyrazole by a dihedral angle of 5.29(9)° so the whole molecular structure is nearly flat (Figure 2).

**Figure 3:**
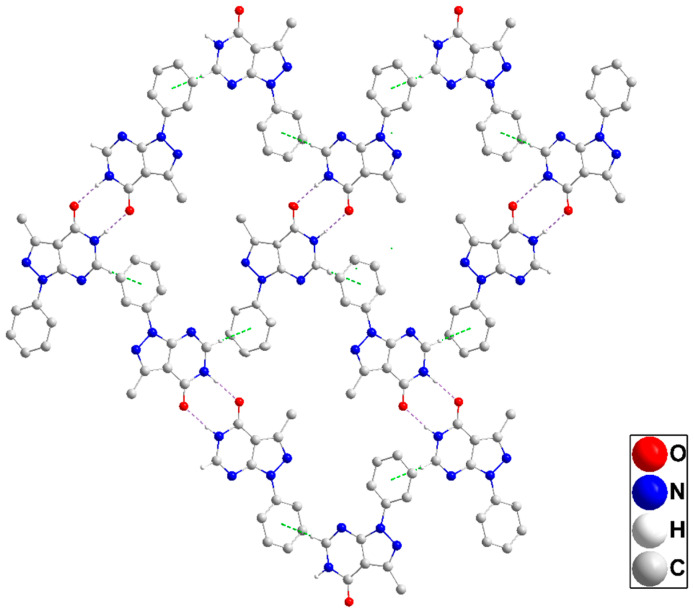
A portion of one layer in **P1** projected onto $\left(10\bar{3}\right),$ with the b-axis horizontal and running from left to right. N—H···O hydrogen bonds and C—H···π(ring) interactions are depicted, respectively, by violet and green dashed lines. Non-interacting hydrogen atoms are omitted for clarity.

In the crystal, inversion dimers are formed by paired N1—H1···O1 hydrogen bonds (Table 1). The dimers are linked by C2—H2···Cg3 interactions (Table 1) to form corrugated layers of molecules parallel to ($10\bar{3}$) (Figure 3). These layers are connected along the *a*-axis direction through slipped π-stacking interactions between phenyl and pyrazole rings (centroid···centroid = 3.5730(8) Å, dihedral angle = 5.29(7)°) with the slippage alternating between 1.02 and 1.33 Å along the stack (Figure S1). Alternatively, the packing can be described as consisting of oblique stacks of molecules connected by the π-stacking interactions which are then joined by the C2—H2···Cg3 interactions. Crystal, data collection, and refinement details are presented in Table S1.

#### 2.1.2. Crystal Structure of 3,5-dimethyl-1-phenyl-1,5-dihydro-4H-pyrazolo[3,4-*d*]pyrimidin-4-one (**P2**)

**Figure 4:**
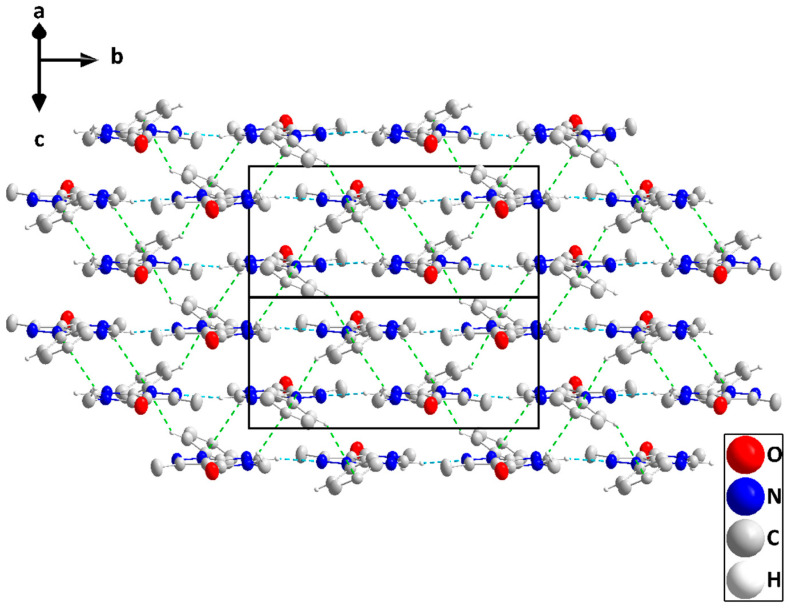
Elevation view of the packing in **P2** seen parallel to ($10\bar{1}$) with C—H···N hydrogen bonds and C—H···π(ring) interactions depicted, respectively, by light blue and green dashed lines.

The pyrazolopyrimidine moiety is planar to within 0.0194(7) Å (rmsd = 0.0150 Å) with the plane of the C8···C13 ring inclined to it by 32.38(4)° (Figure 2). In the crystal, C5—H5···N2 hydrogen bonds (Table 2) form chains of molecules extending along the *b*-axis direction and with the pyrazolopyrimidine moieties approximately parallel to ($10\bar{1}$). These chains stack along the normal to ($10\bar{1}$) with C6—H6A···Cg3 and C9—H9···Cg3 interactions (Table 2) holding them together to form layers (Figure 4). Crystal, data collection, and refinement details are presented in Table S2.

#### 2.1.3. Crystal Structure of 3-methyl-1-phenyl-5-(prop-2-yn-1-yl)-1,5-dihydro-4H-pyrazolo[3,4-*d*]pyrimidinone (**P3**)

**Figure 5:**
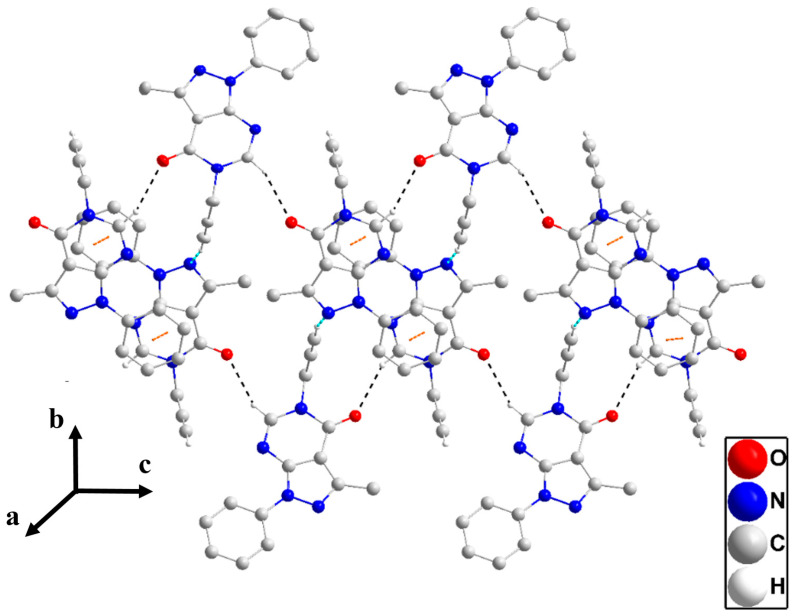
A portion of two chains in **P3** viewed along the *a*-axis direction with C—H···O and C—H···N hydrogen bonds depicted, respectively, by black and light blue dashed lines. The π-stacking interactions are depicted by orange dashed lines.

The dihydropyrazolopyrimidine moiety is not planar as there is a dihedral angle of 4.38(4)° between the mean planes of its constituent rings. The plane, defined by N2/C6/C7/C8, is inclined to the mean plane of the dihydropyrimidine ring by 84.66(8)° indicating the propynyl group is well out of the plane of the ring. The dihedral angle between the mean planes of the dihydropyrimidine and phenyl rings is only 4.38(5)° and this is likely due to the intramolecular C15—H15···N1 hydrogen bond (Table 3 and Figure 2). In the crystal, zigzag chains of molecules extending along the *c*-axis direction are formed by C32—H2···O1 hydrogen bonds (Table 3) and are connected into corrugated layers two molecules thick by C8—H8···N3 hydrogen bonds (Table 3) and π-stacking interactions between phenyl and dihydropyrimidine rings in inversion-related molecules (centroid···centroid (at −*x* + 2, −*y* + 1, −*z* + 1) = 3.6770(8) Å, dihedral angle = 4.91(6)°, slippage = 1.19 Å) (Figure 5). The layers stack along the *a*-axis direction through π-stacking interactions between phenyl and dihydropyrimidine rings in inversion-related molecules (centroid···centroid (at −*x* + 1, −*y* + 1, −*z* + 1) = 3.7177(8) Å, dihedral angle = 4.91(6)°, slippage = 1.66 Å) (Figures S2 and S3). Data collection and refinement details are given in Table S3.

#### 2.1.4. Crystal Structure of 3-methyl-5-(2-oxo-2-phenylethyl)-1-phenyl-1,5-dihydro-4H-pyrazolo[3,4-*d*]pyrimidin-4-one (**P4**)

**Figure 6:**
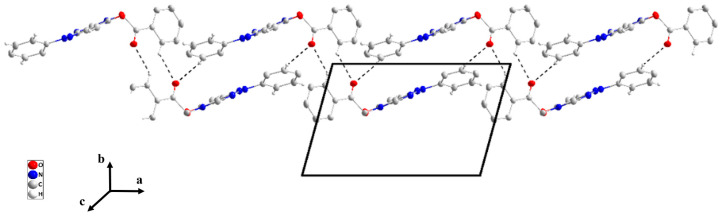
A portion of one chain of **P4** view along the *b*-axis. Dashed lines C—H···O represent hydrogen intermolecular bonding.

The dihydropyrazolopyrimidine moiety is planar to within 0.0102(6) Å (r.m.s. deviation = 0.0087 Å). The mean planes of the C7···C12 and the C15···C20 benzene rings are inclined to the above plane by 29.25(3) and 85.79(3)°, respectively. The C1—N1—C13—C14 torsion angle of −78.99(9)° indicates that the C14=O2 carbonyl group is also nearly perpendicular to the mean plane of the dihydropyrazolopyrimidine moiety (Figure 2). In the crystal, C11—H11···O2 and C20—H20···O2 hydrogen bonds (Table 4) form chains of molecules extending along the *a*-axis direction (Figure 6). The chains are linked by C6—H6C···N2 hydrogen bonds and C9—H9···Cg4 interactions (Table 4) forming layers approximately parallel to (011) (Figure S4). Crystal, data collection, and refinement details are presented in Table S4.

#### 2.1.5. General Structural Comments

In all four structures, the sum of the angles about the nitrogen atom in the pyrazole ring bearing the phenyl substituent is 360° within experimental error, thereby implicating the involvement of the nitrogen lone pair in π bonding with neighboring atoms. As might be expected, this occurs within the five-membered ring. For example, in **P3**, The endocyclic N4—C1 and N4—N3 distances are, respectively, 1.3591(15) and 1.3805(14) Å, while the exocyclic N4—C10 distance is 1.4236(15) Å. Similar patterns are seen in the other structures. A second feature of note is the extent to which the plane of the pendant phenyl group is rotated from the plane of the five-membered ring to which it is attached. In the case of **P3**, it was noted that there is an intramolecular C—H···N hydrogen bond which likely constrains the phenyl group to approach co-planarity with the 5-membered ring but in the others, the dihedral angle is 5.29(9)° in **P1**, 32.38(4)° in **P2,** and 29.25(3)° in **P4**. A survey of 13 related molecules found corresponding dihedral angles ranging from 5.72(18)° in 5-(2-chloroethyl)-1-(4-chlorophenyl)-6-(2-chloropyridin-3-yl)-1,5-dihydro-4*H*-pyrazolo[3,4-*d*]pyrimidin-4-one to 28.96(11)° in 5-(2-chloroethyl)-6-(3-fluorophenyl)-1-phenyl-1,5-dihydro-4*H*-pyrazolo[3,4-*d*]pyrimidin-4-one [20], with the majority among the 13 structures surveyed being between 20 and 27°. Thus, it seems likely that packing considerations are predominant for determining this dihedral angle. If the strongest intermolecular interactions act to keep the dihydropyrazolopyrimidine cores well-separated, then the phenyl ring can rotate out of the plane of the core moiety to relieve intramolecular steric congestion. If not, π-stacking interactions can be influential which will necessitate the five- and six-membered rings being closer to co-planarity.

### 2.2. Optimized Geometries

**Figure 7:**
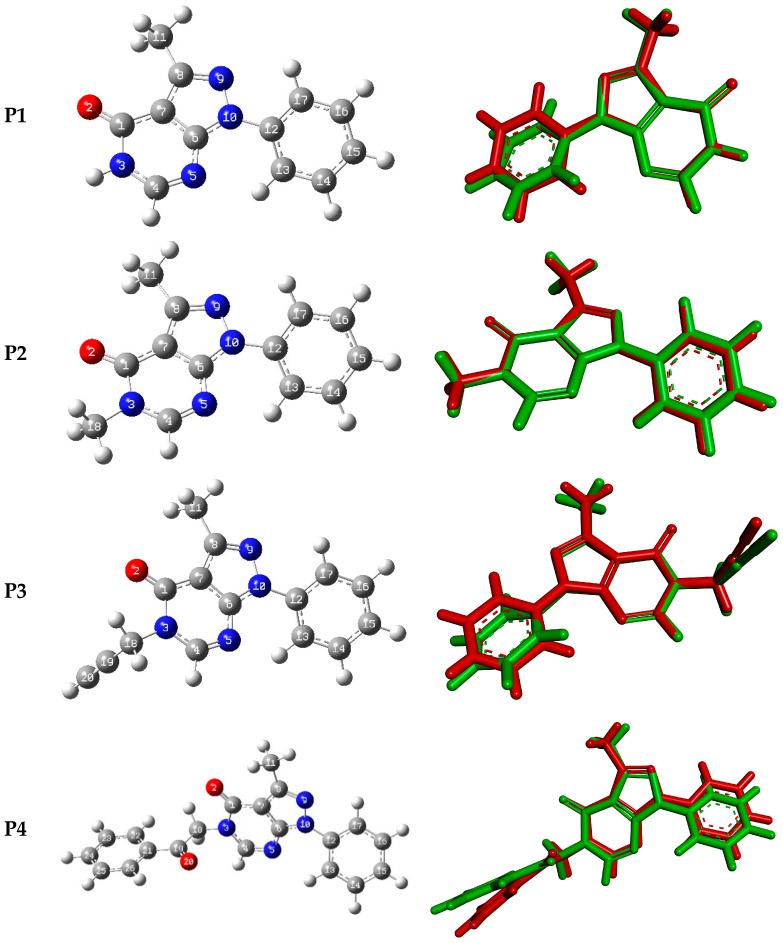
The optimized geometries of **P1**–**P4** (**left**) and their superposition with X-ray-generated ones (**right**, the green color corresponds to the optimized geometry, and the red color corresponds to the X-ray-generated structure).

Figure 7 shows the optimized geometries of **P1**–**P4** along with their corresponding numbering and their superpositions upon the experimental structures. Selected experimental and calculated bond lengths, bond angles, and torsion angles for **P1**–**P4** are summarized in Table 5. The experimental bond lengths are relatively well reproduced with variations less than 0.03 Å. Similarly, the bond angles are relatively well reproduced with variations of less than 3 degrees compared to the experimental ones. The agreement between calculated and experimental torsion angles is much poorer, reflecting the presence of packing constraints in the latter which are absent in the former. RMSD values calculated from the superimposed X-ray and optimized geometries of **P1**–**P4** are 0.33, 0.087 Å, 0.43, and 0.63, respectively. Furthermore, the RMSD values were calculated for bond lengths, bond angles, and dihedral angles of **P1**–**P4**, and they are found in ranges of 0.08–0.12Å, 0.50–5.00 degrees, and 3.50–13.00 degrees, respectively. Figure S13 displays the linear regression curves between the experimental bond lengths, bond angles, and dihedral angles, and their corresponding predicted ones. The correlation between the experimental bond lengths, bond angles, and torsion angles with their corresponding calculated ones yield linear curves of correlation coefficients in the ranges of 92.00–99.50%, 91.50–99.95%, and 99.00–99.90%, respectively (Figure S13). 

### 2.3. Hirshfeld Surface Analysis

**Figure 8:**
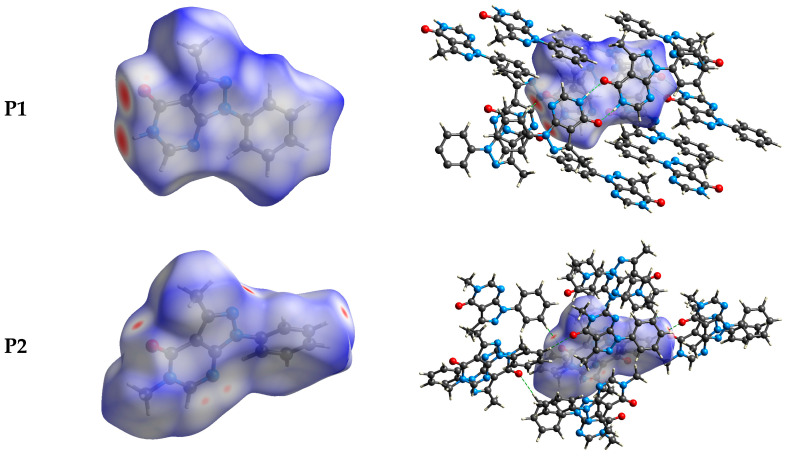

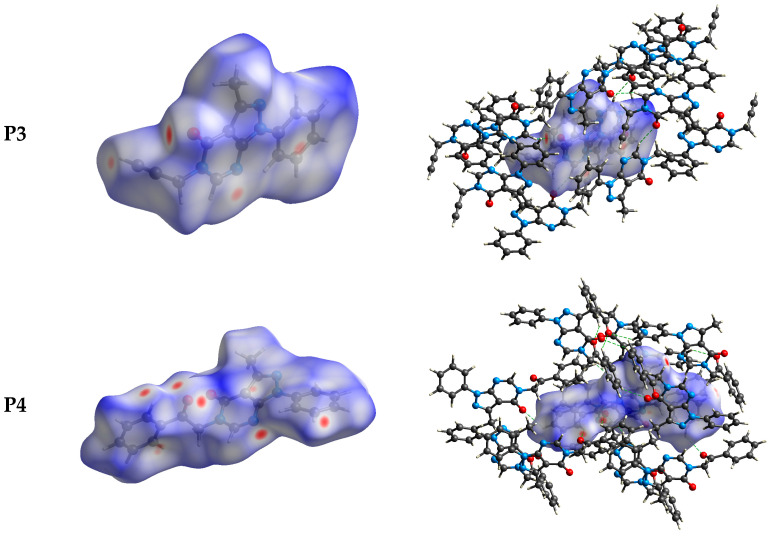
The d_norm_ surfaces for viewing hydrogen bonding interactions in **P1**–**P4** crystals.

**Figure 9:**
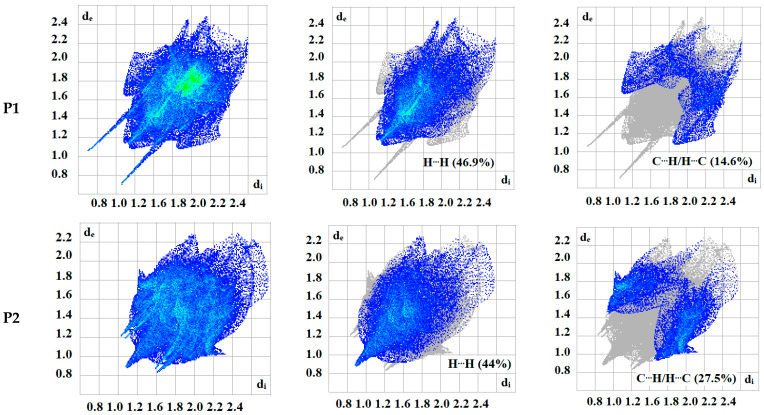

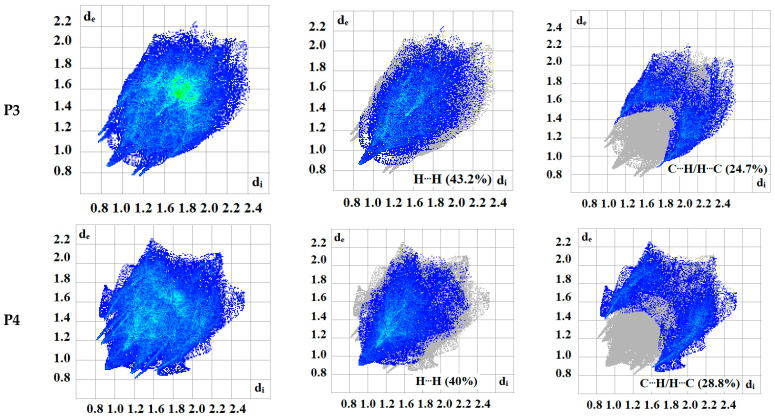
2D fingerprints of the highest intercontacts in **P1**–**P4**.

The intermolecular bonding interactions in **P1**–**P4** crystals were investigated by analyzing their corresponding Hirshfeld surfaces (HS). Descriptions and interpretations of the plots obtained from the HS analysis have been published [21]. The red spots on the Hirshfeld surface indicate contacts less than the sum of the van der Waals radii of the respective elements, while those longer than this sum are displayed in blue, and those on the order of this sum are shown in white (Figure 8, left column). The right column of Figure 8 shows each molecule with its HS surrounded by several “next neighbors” that are involved in close contact. The 2D fingerprints giving the contacts contributing most to the overall intermolecular interactions in **P1**–**P4** are displayed in Figure 9. In all crystals, the highest intercontacts are found for H···H, and C···H/H···C interactions (Table 6).

This can be attributed to the significant hydrogen content of the four molecules and the fact that most of the hydrogen atoms are attached to phenyl and methyl groups which protrude out from the centers of the molecules and thus comprise a significant portion of their peripheries. Although not making the largest intermolecular contacts, the fingerprint plot for **P1** is notable for the pair of sharp spikes with d_e_ + d_i_ ≈ 2.1 Å. These can be attributed to the N—H···O hydrogen bonds which, in addition to being the shortest intermolecular contact, have essentially the same H···O distance throughout the crystal. For the other three molecules, the fingerprint plots show two pairs of sharp points that extend slightly from either side of the center point. Those in **P1** both have d_e_ + d_i_ ≈ 2.4 Å which is consistent with them representing the C—H···N hydrogen bonds. As the H···N contact distance is longer than the H···O distance in **P1**, and because it is closer in magnitude to the other intermolecular contacts in **P2**, these peaks are much less prominent. The same comment applies to the other two overall fingerprint plots with **P3** showing peaks at d_e_ + d_i_ ≈ 2.36 and 2.20 Å and **P4** having them at 2.40 and 2.36 Å. In both cases, these can be attributed to C—H···N and C—H···O hydrogen bonds, respectively.

### 2.4. Antiproliferative Activity

**Figure 10:**
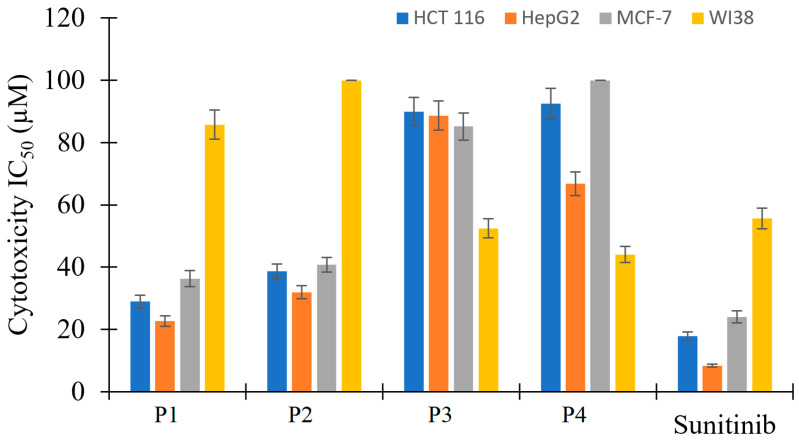
Bar representation of the in vitro antiproliferative activity of **P1**–**P4** and sunitinib against HCT 116, HepG2, and MCF-7 cancer cell lines, and one normal cell line WI38.

The antiproliferative activity of **P1**–**P4** was evaluated by the MTT standard assay and sunitinib (a well-known FDA-approved multi-kinase inhibitor) as a reference against HCT 116, HepG2, and MCF-7 cell lines, and WI38 normal cell line. Compounds **P3** and **P4** exhibited very weak anticancer activity with IC_50_ values above 67 µM (Table 7 and Figure 10); they show more cytotoxicity toward the normal cell line than towards the cancer cell lines. By contrast, **P1** and **P2** showed an antiproliferative activity comparable to the reference standard anticancer drug against the cancer cell lines as their results have less than three-fold difference compared to sunitinib (IC_50_ values in 22.7–40.75 µM). **P1** and **P2** showed excellent selectivity indices as they displayed very weak cytotoxic effects on the normal cell line. The obtained results indicate that biological targets through which the tested compounds exhibit their anticancer effect could not perfectly accommodate relatively huge substitutions on position number three. Therefore, **P3** and **P4** exhibited weak anticancer effects.

### 2.5. Molecular Docking Results

Since **P1**–**P4** compounds have no better antiproliferative activity than the reference, their binding affinity within the binding site of the human DNA topoisomerase was explored using molecular docking to better understand their modest activity. The calculated binding energies of the stable complexes **P_i=1–4_**-DNA topoisomerase, the number of intermolecular hydrogen bonds, and the number of closest amino acids that interact with **P1**–**P4** are summarized in Table 8.

**Figure 11:**
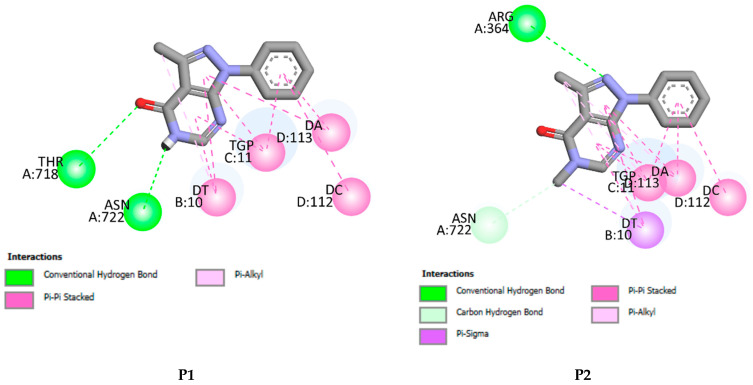

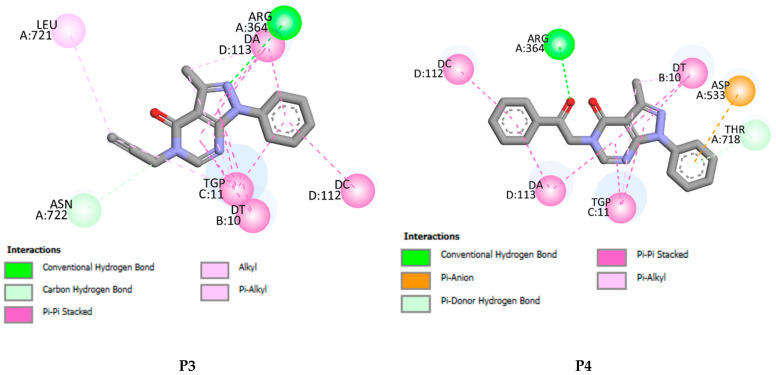
2D binding affinities of **P1**–**P4** into the DNA topoisomerase binding site.

**Figure 12:**
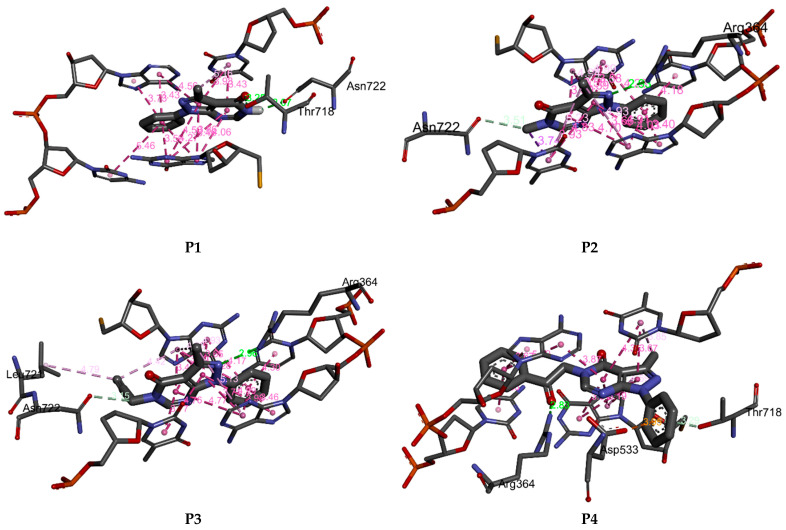
3D binding affinities of **P1**–**P4** into the DNA topoisomerase binding site.

**P1**–**P4** fit well into the DNA topoisomerase binding site. They lead to the formation of stable complexes with binding energies in the range of −7 to −8.52 kcal.mol^−1^. The negative binding energies may indicate that the inhibition of DNA topoisomerase by **P1**–**P4** is thermodynamically favorable and spontaneous. Figure 11 and Figure 12 display, respectively, the 2D and 3D binding interaction modes established in stable **P_i=1–4_**-DNA topoisomerase complexes. These interactions involve (i) conventional hydrogen bond interactions (ii) π-π stacked interactions, (iii) π-alkyl, (iv) π-σ, and (v-carbon hydrogen bond interactions (Figure 11 and Figure 12). Binding energies of stable **P_i=1–4_**-DNA topoisomerase complexes vary slightly (less than 1.5 kcal-mol^−1^) so the binding energy may not be a significant factor in explaining the observed antiproliferative activity of **P1**–**P4** against the three cancer cell lines. Among **P1**–**P4**, **P1** shows the best activity against the three cell lines and it may be that the higher activity of **P1** relates to the number of hydrogen bonds it forms with the amino acids of DNA topoisomerase and the types of amino acids involved. In **P2**–**P4**, a hydrogen bond is established with ARG A364. However, in **P1**, its keto and amine functional groups form two hydrogen bonds with THR A178 and ASN A722 of DNA topoisomerase of distances 3.25 and 2.07 Å, respectively (Figure 11 and Figure 12). Thus, one may conclude that the higher inhibition of **P1** may return to hydrogen binding established between ASN A722 and the amine of pyrazolo[3,4-*d*]pyrimidine.

## 3. Materials and Methods

### 3.1. Reagents and Instruments

All chemicals used in this work were purchased from commercial sources (Sigma-Aldrich, Fluka, Saint Louis, MO, USA). They were used without further purification. Mel-Temp 3.0 apparatus was used to determine the melting points. NMR spectra were generated using Bruker Advance/DPX 250 (250 MHz ^1^H, 62.9MHz ^13^C) and Bruker (Billerica, MA, USA) Advance II/DPX 400 (400 MHz ^1^H, 100 MHz ^13^C) spectrometers.

### 3.2. Synthesis

**Scheme 1:**
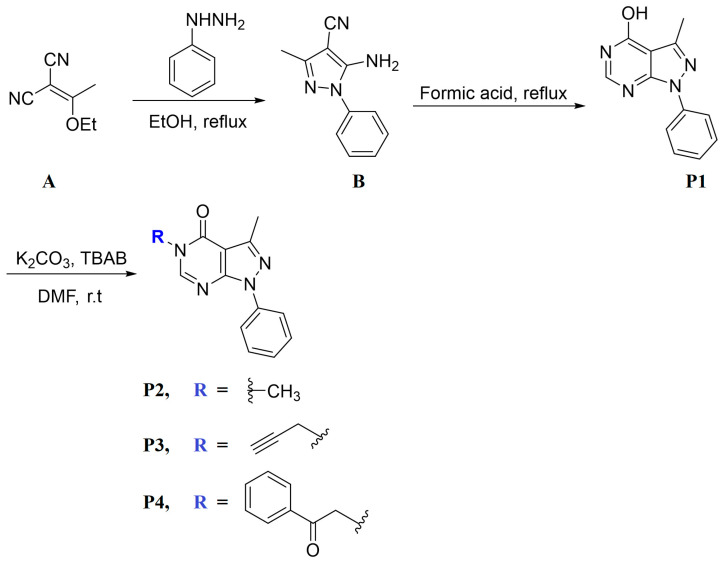
Synthesis path of **P1**–**P4**.

Pyrazolo [3,4-*d*] pyrimidine derivatives (**P1**–**P4**) were synthesized by following the procedure mentioned under Scheme 1. Based on the literature, 2-(1-ethoxyethylidene)malononitrile (**A**) and phenyl hydrazine were refluxed in ethanol for 2 h to afford 5-amino-3-methyl-1-phenyl-1H-pyrazole-4-carbonitrile (**B**) [20]. The latter was reacted with formic acid under reflux for 7 h to give compound **P1** in good yield, which was reacted with the selected alkylating agent: methyl iodide, propargyl bromide, phenacyl bromide to give the final products **P2**, **P3**, and **P4**, respectively, in good yield.

#### 3.2.1. Synthesis of **P1**

A solution of 5-amino-3-methyl-1-phenyl-1*H*-pyrazole-4-carbonitrile (**B**) (1.0 g, 5.04 mmol) in formic acid (30 mL) was refluxed for 7 h. The final mixture was poured into ice water and the resulting precipitate was filtered off, dried, and recrystallized from ethanol (Scheme 1).

Yield: 83%. mp: 153–155 °C. ^1^H NMR (400 MHz, DMSO-*d*_6_) δ 12.34 (s, 1H), 8.13 (s, 1H), 8.03–8.01 (m, 2H), 7.54–7.50 (m, 2H), 7.37–7.33 (m, 1H), 2.52 (s, 3H) (Figure S5).^13^C NMR (101 MHz, DMSO-*d*_6_) δ 157.99, 152.24, 148.88, 145.93, 138.29, 129.11, 126.62, 121.36, 105.74, 13.37 (Figure S6).

#### 3.2.2. Synthesis of **P2**–**P4**

A DMF (5 mL) solution containing **P1** (0.1 g, 0.44 mmol), K_2_CO_3_ (1.2 equiv.), and tetra *n*-butylammonium bromide (TBAB) catalyst was stirred for 10 min. Then, the selected alkylating agent (1.2 equiv.) was added to the solution, and the mixture was stirred overnight. The mixture was then poured into ice water and the resulting precipitate was filtered off, dried, and recrystallized from ethanol yielding the final product (Scheme 1).

3,5-Dimethyl-1-phenyl-1,5-dihydro-4*H*-pyrazolo[3,4-*d*]pyrimidin-4-one (**P2**)

Yield: 70%. mp: 143–145 °C. ^1^H NMR (400 MHz, DMSO-*d*_6_) δ 8.40 (s, 1H), 8.02–7.99 (m, 2H), 7.54–7.50 (m, 2H), 7.37–7.33 (m, 1H), 3.47 (s, 3H), 2.52 (s, 3H) (Figure S7).^13^C NMR (101 MHz, DMSO-*d*_6_) δ 157.60, 151.90, 151.70, 145.86, 138.27, 129.24, 126.66, 121.20, 104.94, 33.15, 13.41 (Figure S8).

3-Methyl-1-phenyl-5-(prop-2-yn-1-yl)-1,5-dihydro-4*H*-pyrazolo[3,4-*d*]pyrimidin-4-one (**P3**)

Yield 65%, mp = 155–157 °C. ^1^H NMR (250 MHz, DMSO-*d*_6_) δ 8.54 (s, 1H), 8.02–7.99 (m, 2H), 7.54 (m, 2H), 7.39 (m, 1H), 4.82 (d, *J* = 2.5 Hz, 2H), 3.43 (t, *J* = 2.5 Hz, 1H), 2.55 (s, 3H) (Figure S9).^13^C NMR (63 MHz, DMSO-*d*_6_) δ 156.31, 151.44, 150.71, 146.10, 138.02, 129.21, 126.87, 121.47, 104.84, 78.65, 75.75, 34.63, 13.34 (Figure S10).

3-Methyl-5-(2-oxo-2-phenylethyl)-1-phenyl-1,5-dihydro-4*H*-pyrazolo[3,4-*d*]pyrimidin-4-one (**P4**)

Yield 69%, mp = 1170–172 °C. ^1^H NMR (250 MHz, DMSO-*d*_6_) δ 8.43 (s, 1H), 8.12–8.03 (m, 4H), 7.75–7.73 (m, 1H), 7.65–7.53 (m, 4H), 7.42–7.35 (m, 1H), 5.64 (s, 2H), 2.53 (s, 3H) (Figure S11).^13^C NMR (63 MHz, DMSO-*d*_6_) δ 192.84, 156.91, 151.84, 151.71, 146.07, 138.12, 134.27, 129.23, 129.07, 128.10, 126.82, 121.44, 104.85, 51.48, 13.32 (Figure S12).

**Scheme 2:**
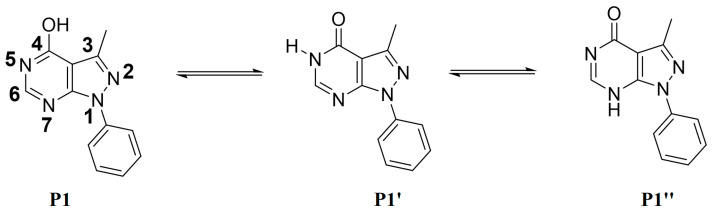
Tautomerism of compound **P1**.

The possible tautomeric forms of **P1** (**P1**–**P1″**) are displayed in Scheme 2. It possesses three reactive sites towards the alkylating agents N5, N7, and O of OH at C4. The alkylation reactions carried out under liquid–solid phase transfer catalysis lead in each case to only one alkylated product, **P2**–**P4**, showing that N5 of the pyrazolopyrimidine is the most reactive site of the heterocyclic compound **P1**.

### 3.3. Single Crystal X-Ray Diffraction

The crystal structures of **P1**–**P4** were generated from X-ray intensity data collected on a Bruker D8 QUEST PHOTON 3 diffractometer. More details on the X-ray diffraction methodology may be found in our previous studies [21].

### 3.4. Hirshfeld Surface Study

Hirshfeld surfaces and fingerprint plots of **P1**–**P4** were generated with Crystal Explorer 3.0 [22,23]. d_norm_ was mapped from −0.60 a.u (blue) to 1.25 a.u (red), while 2D fingerprints were plotted and displayed on a 0.8–2.4 Å expanded scale.

### 3.5. DFT Calculations

The stable geometries of **P1**–**P4** were optimized in solvent using the B3LYP hybrid functional along with the basis set 6–311++G(d,p). The frequency calculations confirm that the optimized geometries of **P1**–**P4** are true minima. The experimental atom coordinates of **P1**–**P4** were used as starting inputs for the optimization. The solvent was modulated using the implicit polarizable continuum model (PCM) and chloroform as solvent [24]. Theoretical calculations were accomplished using the Gaussian 16 software [25].

### 3.6. Molecular Docking Study

The binding affinities of **P1**–**P4** into the human DNA topoisomerase binding site as the target of anticancer agents were explored with the Autodock package [26]. The structural geometries of the human topoisomerase I-DNA and its EHD ligand were downloaded from the data website RCSB (PDB file 1T8I) [27]. The re-docking of EHD is relatively well reproduced, with an RMSD value of 0.30 Å and binding energy of −10.50 kcal-mol^−1^. Details of the docking calculations may be found in our previous study [21].

### 3.7. Cytotoxicity Assay

The antiproliferative activity of **P1**–**P4** was assessed by using an MTT standard assay and sunitinib drug towards HCT 116, HepG2, MCF-7 cells, and WI38 normal cell line using the previously described methodology [21].

## 4. Summary

Four new pyrazolo[3,4-*d*]pyrimidines (**P1**–**P4**) have been synthesized and their structures determined using NMR spectroscopy and single crystal X-ray diffraction. Hirshfeld surfaces and 2D fingerprints revealed that H···H and C···H/H···C are the major intermolecular contacts in **P1**–**P4**. Bond lengths, bond angles, and torsion angles of **P1**–**P4** are relatively well reproduced at the B3LYP level of theory with correlation coefficients higher than 90%. The antiproliferative activity of **P1**–**P4** is weak compared to the antiproliferative activity of the reference standard anticancer drug against the cancer cells with IC_50_ of 22.7–40.75 µM. Molecular docking reveals that DNA topoisomerase inhibition by **P1**–**P4** is a spontaneous, thermodynamically favorable process and is influenced by the number of hydrogen bonds and the types of amino acids involved in the formation of hydrogen bonds between **P1**–**P4** and the amino acids of DNA topoisomerase.

## Figures and Tables

**Table 1:** Hydrogen bond geometry (Å, °) for **P1**. Cg3 is the centroid of the C6···C11 benzene ring.

*D*—H···*A*	*D*—H	H···*A*	*D*···*A*	*D*—H···*A*
N1—H1···O1 ^i^	0.917 (9)	1.877 (9)	2.7912 (13)	174.9 (14)
C2—H2···Cg3 ^ii^	0.95	2.86	3.7328 (14)	154
C7—H7···N2	0.95	2.30	2.9746 (17)	127

Symmetry codes: (i) −x + 3, −y + 1, −z + 1; (ii) −x + 3/2, y + 1/2, −z + 1/2.

**Table 2:** Hydrogen bond geometry (Å, °) for **P2**. Cg3 is the centroid of the C8···C13 benzene ring.

*D*—H···*A*	*D*—H	H···*A*	*D*···*A*	*D*—H···*A*
C5—H5···N2 ^i^	0.95	2.57	3.4517 (17)	154
C6—H6A···Cg3 ^ii^	0.98	2.92	3.5166 (19)	121
C9—H9···Cg3 ^iii^	0.95	2.99	3.7890 (19)	142

Symmetry codes: (i) −*x* + 1, *y* + 1/2, −*z* + 3/2; (ii) −*x* + 1, −*y* + 1, −*z* + 2; (iii) *x*, −*y* − 1/2, *z* − 1/2.

**Table 3:** Hydrogen bonding geometry (Å, °) for **P3**.

*D*—H···*A*	*D*—H	H···*A*	*D*···*A*	*D*—H···*A*
C2—H2···O1 ^i^	0.95	2.42	3.3115 (15)	156
C8—H8···N3 ^ii^	0.95	2.48	3.3915 (18)	160
C15—H15···N1	0.95	2.28	2.9506 (17)	127

Symmetry codes: (i) *x*, −*y* + 1/2, *z* + 1/2; (ii) −*x* + 2, *y* − 1/2, −*z* + 1/2.

**Table 4:** Hydrogen bond geometry (Å, °) for **P4**. Cg4 is the centroid of the C15···C20 benzene ring.

*D*—H···*A*	*D*—H	H···*A*	*D*···*A*	*D*—H···*A*
C6—H6C···N2 ^i^	0.96	2.56	3.5153 (12)	178
C9—H9···Cg4 ^i^	0.93	2.68	3.5863 (12)	166
C11—H11···O2 ^ii^	0.93	2.55	3.2895 (13)	137
C20—H20···O2 ^iii^	0.93	2.55	3.2895 (13)	137

Symmetry codes: (i) −*x* + 1, *y* − 1/2, −*z* + 1/2; (ii) −*x* + 1, −*y* + 1, −*z*; (iii) −*x*, −*y* + 1, −*z.*

**Table 5:** Selected experimental and calculated geometrical parameters of **P1**–**P4**.

	P1			P2			P3			P4		
	X-Ray	Cal	ΔX ^a^	X-Ray	Cal	ΔX ^a^	X-Ray	Cal	ΔX ^a^	X-Ray	Cal	ΔX ^a^
Bond length, B(Å)												
C1-O1	1.239	1.224	0.01	1.220	1.225	0.00	1.225	1.223	0.00	1.220	1.225	0.00
C1-N3	1.392	1.418	0.03	1.407	1.427	0.02	1.417	1.431	0.01	1.424	1.430	0.01
N9-N10	1.387	1.376	0.01	1.378	1.377	0.00	1.381	1.376	0.00	1.385	1.376	0.01
N10-C12	1.425	1.425	0.00	1.415	1.424	0.01	1.424	1.424	0.00	1.420	1.424	0.00
N3-C18	-	-	-	1.465	1.468	0.00	1.479	1.477	0.00	1.458	1.457	0.00
C18-C19	-	-	-	-	-	-	1.466	1.462	0.00	1.528	1.538	0.01
C19-O20	-	-	-	-	-	-	-	-	-	1.215	1.216	0.00
Bond angles, A(°)												
N3-C1-C7	111.90	110.46	1.45	111.81	111.65	0.16	111.25	111.24	0.01	111.23	111.43	0.20
N5-C6-N10	126.72	126.56	0.16	126.17	127.07	0.90	125.89	126.92	1.03	126.31	126.94	0.64
C1-N3-C18	-	-	-	117.27	117.01	0.26	117.96	117.75	0.21	117.10	117.52	0.42
N3-C18-C19	-	-	-	-	-	0.00	110.60	112.92	2.32	111.20	112.17	0.98
C18-C19-C20	-	-	-	-	-	0.00	176.61	179.33	2.72			0.00
C18-C19-O20	-	-	-	-	-	-	-	-	-	119.79	119.99	0.20
Torsion angles, D(°)												
O2-C1-C7-C8	−2.35	1.19	3.54	2.63	1.26	1.37	−5.60	1.21	6.81	−0.20	−1.40	1.21
N5-C6-N10-C12	2.58	−0.12	2.71	0.09	−0.54	0.63	−0.15	−0.64	0.49	4.43	0.62	3.81
N9-N10-C12-C17	6.02	33.01	26.99	31.34	30.62	0.72	5.51	31.55	26.04	−27.06	−32.12	5.05
O2-C1-N3-C18	-	-	-	−2.24	0.02	2.26	3.72	0.96	2.75	−3.93	−2.66	1.27
C1-N3-C18-C19	-	-	-	-	-	0.00	82.62	91.88	9.26	−78.99	−90.41	11.41
N3-C18-C19-O20	-	-	-	-	-	-	-	-	-	5.07	−6.01	11.08
C18-C19-C21-C22	−2.35	1.19	3.54	2.63	1.26	1.37	−5.60	1.21	6.81	−0.20	−1.40	1.21

^a^ X = B, A or D, where B is for B (bond length), A for bond angle, and D for torsion angle.

**Table 6:** The largest contributors to intermolecular contacts in **P1**–**P4**.

	P1	P2	P3	P4
H···H	46.9	44.0	43.2	40.0
C···H/H···C	14.6	27.5	24.7	28.8
O···H/H···O	10.0	11.1	10.0	15.2
N···H/H···N	10.8	11.5	6.7	8.7

**Table 7:** In vitro antiproliferative activity of **P1**–**P4** against HCT 116, HepG2, and MCF-7 cancer cell lines, and a normal cell line WI38.

Compound	In Vitro Cytotoxicity IC_50_ (µM) *				Selectivity Index
	HCT 116	HepG2	MCF-7	WI38	
**P1**	28.93 ± 2.1	22.70 ± 1.7	36.32 ± 2.6	85.73 ± 4.7	0.34
**P2**	38.68 ± 2.3	31.93 ± 2.1	40.75 ± 2.4	>100	0.37
**P3**	89.92 ± 4.5	88.62 ± 4.7	85.16 ± 4.4	52.49 ± 3.1	1.67
**P4**	92.47 ± 4.9	66.81 ± 3.8	>100	44.03 ± 2.6	1.96
Sunitinib	17.91 ± 1.3	8.38 ± 0.5	24.06 ± 2.0	55.63 ± 3.3	0.30

* Results indicate three different experiments.

**Table 8:** Calculated binding energies (BE), number of hydrogen bonds (HBs), and the number of closest residues (NR) to the docked **P1**–**P4** into DNA topoisomerase binding site.

	BE (kcal/mol)	HBs	NR ^1^
**P1**	−7.01	2	ASN A722, THR A718, DT B10, TGP C11, DA D113, DC D112
**P2**	−7.03	1	ARG A364, ASN A722, DT B10, TGP C11, DA D113, DC D112
**P3**	−7.51	1	ARG A364, ASN A722, LEU A721, DT B10, TGP C11, DA D113, DC D112
**P4**	−8.52	1	ARG A364, TYR A426, LYS A425, DT B10, TGP C11, DA D113, DC D112

^1^ Number of amino acids of DNA Topoisomerase that interact with **P1**–**P4**.

## Data Availability

Data are contained within the article and supplementary materials.

## Supplementary Materials

The following supporting information can be downloaded at: https://www.mdpi.com/article/10.3390/molecules29215020/s1, Figure S1: Perspective view of the packing with N—H···O hydrogen bonds and C—H···π(ring) and π-stacking interactions depicted, respectively, by violet, green, and orange dashed lines. Non-interacting hydrogen atoms are omitted for clarity; Figure S2: Packing viewed along the *a*-axis direction with intermolecular interactions depicted as in Figure 4; Figure S3: Packing viewed along the *c*-axis direction with intermolecular interactions depicted as in Figure 4; Figure S4: Packing viewed along the *b*-axis direction. C—H···O and C—H···N hydrogen bonds are depicted, respectively, by black and light blue dashed lines while C—H···π(ring) interactions are depicted by green dashed lines. Non-interacting hydrogen atoms are omitted for clarity; Figure S5: ^1^H NMR spectrum of compound **D**; Figure S6: ^13^C NMR spectrum of compound **D**; Figure S7: ^1^H NMR spectrum of compound **D3**; Figure S8: ^13^C NMR spectrum of compound **D3**; Figure S9: ^1^H NMR spectrum of compound **D4**; Figure S10: ^13^C NMR spectrum of compound **D4**; Figure S11: ^1^H NMR spectrum of compound **D5**; Figure S12: ^13^C NMR spectrum of compound **D5**; Figure S13: Correlation curves between experimental and calculated bond lengths, bond angles, and dihedral angles of P1-P4; Table S1: The crystal data and structure refinement details of compound **D**; Table S2: The crystal data and structure refinement details of compound **D3**; Table S3: The crystal data and structure refinement details of compound **D4**; Table S4: The crystal data and structure refinement details of compound **D5**.
