# Supplementary material for: Synthesis of New Pyrazolo[3,4-d]pyrimidine Derivatives: NMR Spectroscopic Characterization, X-Ray, Hirshfeld Surface Analysis, DFT, Molecular Docking, and Antiproliferative Activity Investigations
Source: Molecules. 2024 Oct 24;29(21):5020. doi: 10.3390/molecules29215020 (PMC11547452; doi:10.3390/molecules29215020)
Supplement: Supplementary file 1 [file molecules-29-05020-s001.zip › molecules-3246050-supplementary.pdf]

molecules

# Synthesis of New Pyrazolo[3,4-*d*]pyrimidine Derivatives: NMR Spectroscopic Characterization, X-Ray, Hirshfeld Surface Analysis, DFT, Molecular Docking, and Antiproliferative Activity Investigations

**Mohamed El Hafi [1,2], El Hassane Anouar [3,*], Sanae Lahmidi [2], Mohammed Boulhaoua [2,4], Mohammed Loubidi [5], Ashwag S. Alanazi [6], Insaf Filali [3], Mohamed Hefnawy [7], Lhoussaine El Ghayati [2], Joel T. Mague [8] and El Mokhtar Essassi [2]**

[1] Faculty of Medicine and Pharmacy, Mohammed First University, Oujda 60000, Morocco; elhafi.mohamed1@gmail.com
[2] Laboratory of Heterocyclic Organic Chemistry, Department of Chemistry, Faculty of Sciences, Mohammed V University in Rabat, Rabat 10106, Morocco; lahmidi_sanae@yahoo.fr (S.L.); mboulhaoua@gmail.com (M.B.); lelghayati2@gmail.com (L.E.G.); emessassi@yahoo.fr (E.M.E.)
[3] Department of Chemistry, College of Science and Humanities in Al-Kharj, Prince Sattam bin Abdulaziz University, Al-Kharj 11942, Saudi Arabia; insaf_filali@yahoo.fr
[4] Institute of Chemistry, ELTE Eötvös Loránd University, Pázmány P. sétány 1/A, 1117 Budapest, Hungary; mboulhaoua@gmail.com
[5] Department of Chemistry, Faculty of Sciences Semlalia, Cadi Ayyad University, Marrakech 40000, Morocco; m.loubidi@gmail.com
[6] Department of Pharmaceutical Sciences, College of Pharmacy, Princess Nourah bint Abdulrahman University, Riyadh 11671, Saudi Arabia; asalanzi@pnu.edu.sa
[7] Department of Pharmaceutical Chemistry, College of Pharmacy, King Saud University, Riyadh 11451, Saudi Arabia; mhefnawy@ksu.edu.sa
[8] Department of Chemistry, Tulane University, New Orleans, LA 70118, USA; joelt@tulane.edu
* Correspondence: anouarelhassane@yahoo.fr

molecules

MDPI

**Figure S1**

Perspective view of the packing with N—H···O hydrogen bonds and C—H···π(ring) and π-stacking interactions depicted, respectively, by violet, green, and orange dashed lines. Non-interacting hydrogen atoms are omitted for clarity.

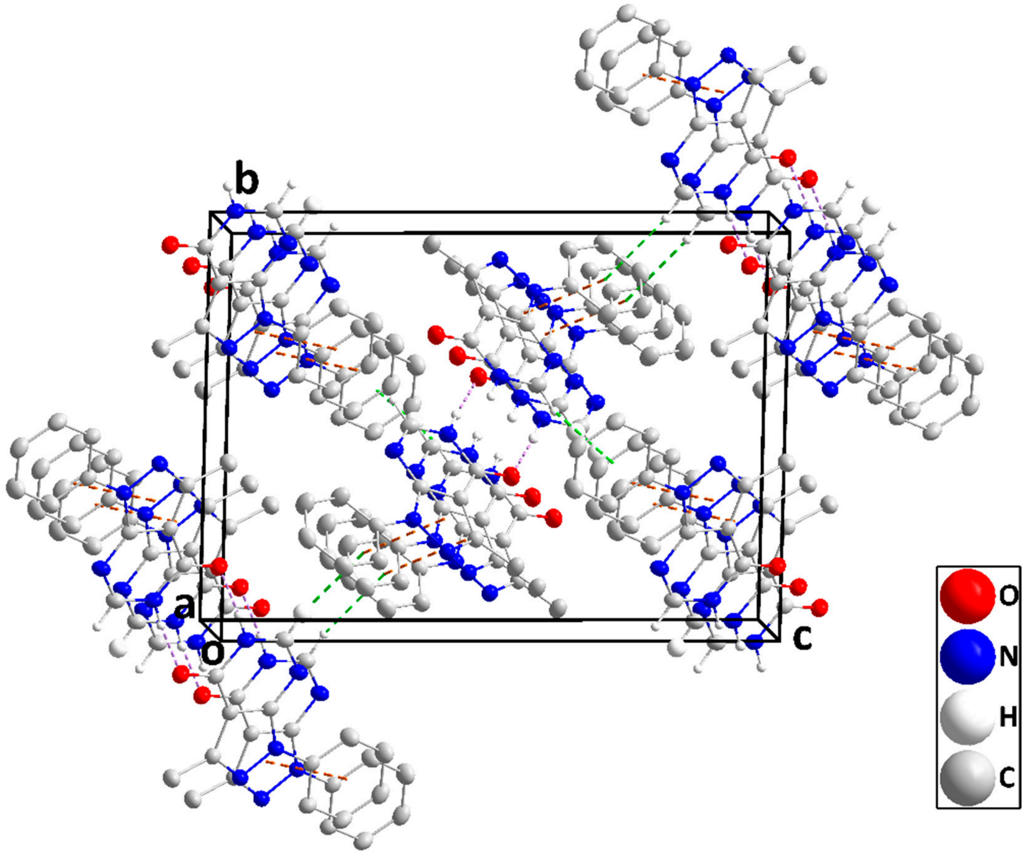

molecules MDPI

**Figure S2**

Packing viewed along the *a*-axis direction with intermolecular interactions depicted in Figure 4.

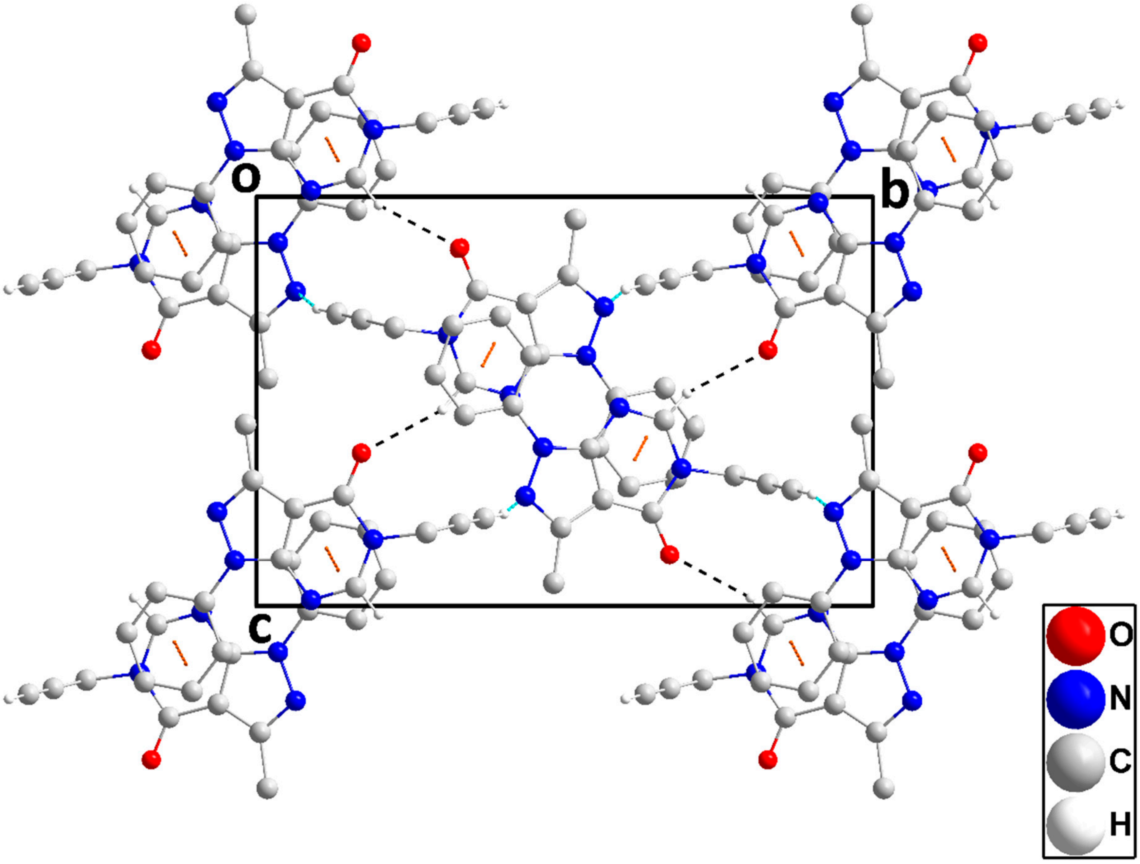

molecules

MDPI

**Figure S3**

Packing viewed along the *c*-axis direction with intermolecular interactions depicted in Figure 4.

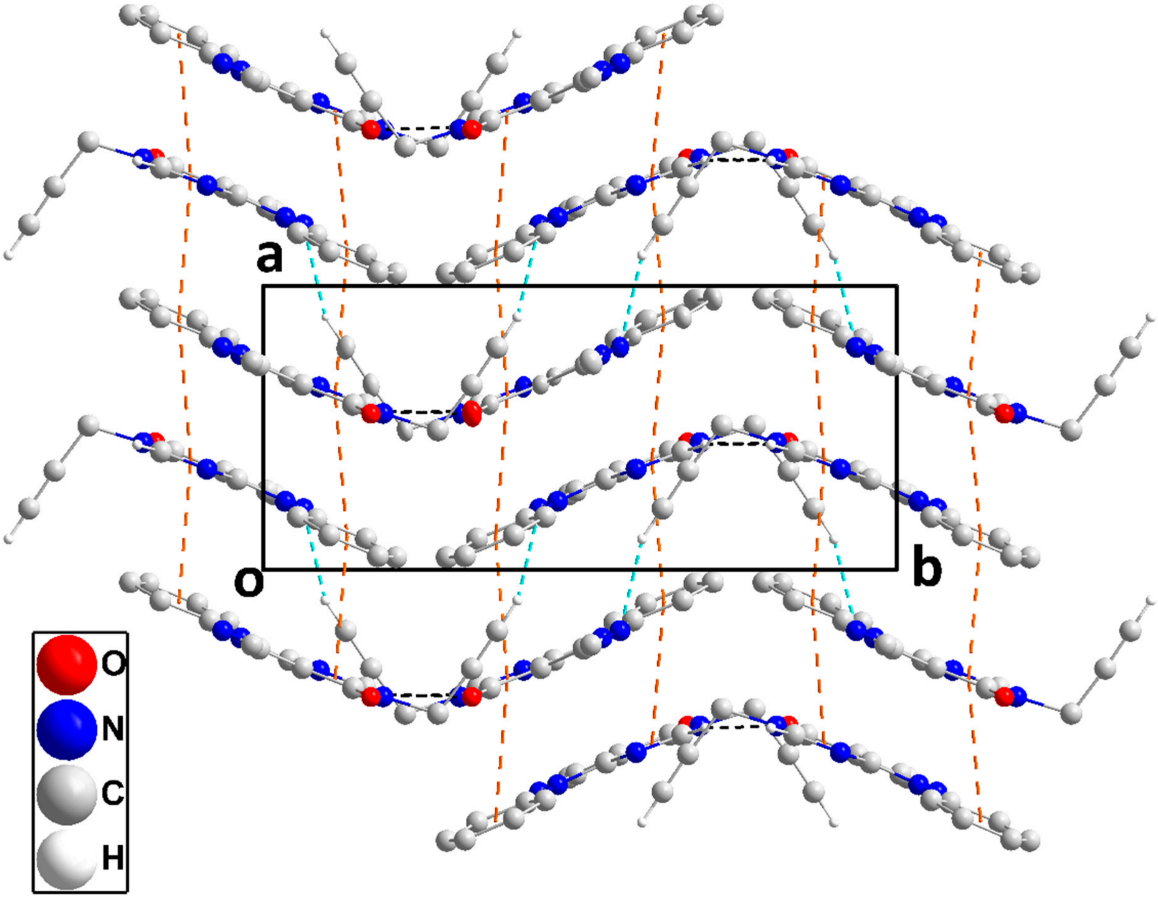

molecules MDPI

**Figure S4**

Packing viewed along the *b*-axis direction. C—H···O and C—H···N hydrogen bonds are depicted, respectively, by black and light blue dashed lines while C—H···π(ring) interactions are depicted by green dashed lines. Non-interacting hydrogen atoms are omitted for clarity.

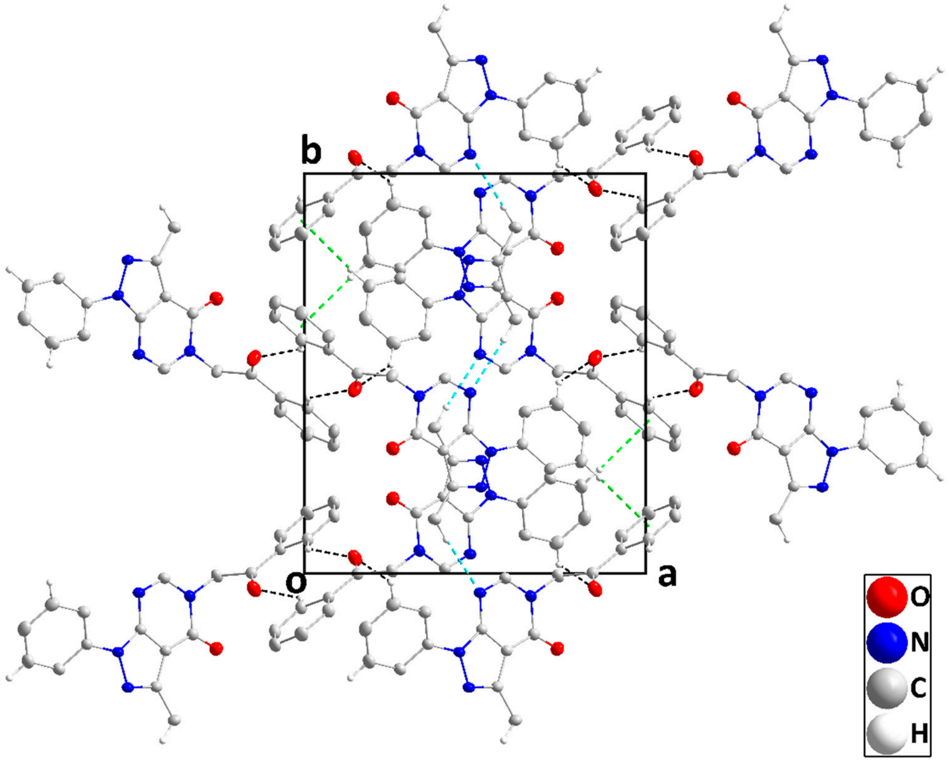

molecules

MDPI

**Figure S5**

$^{1}$H NMR spectrum of compound **P1**.

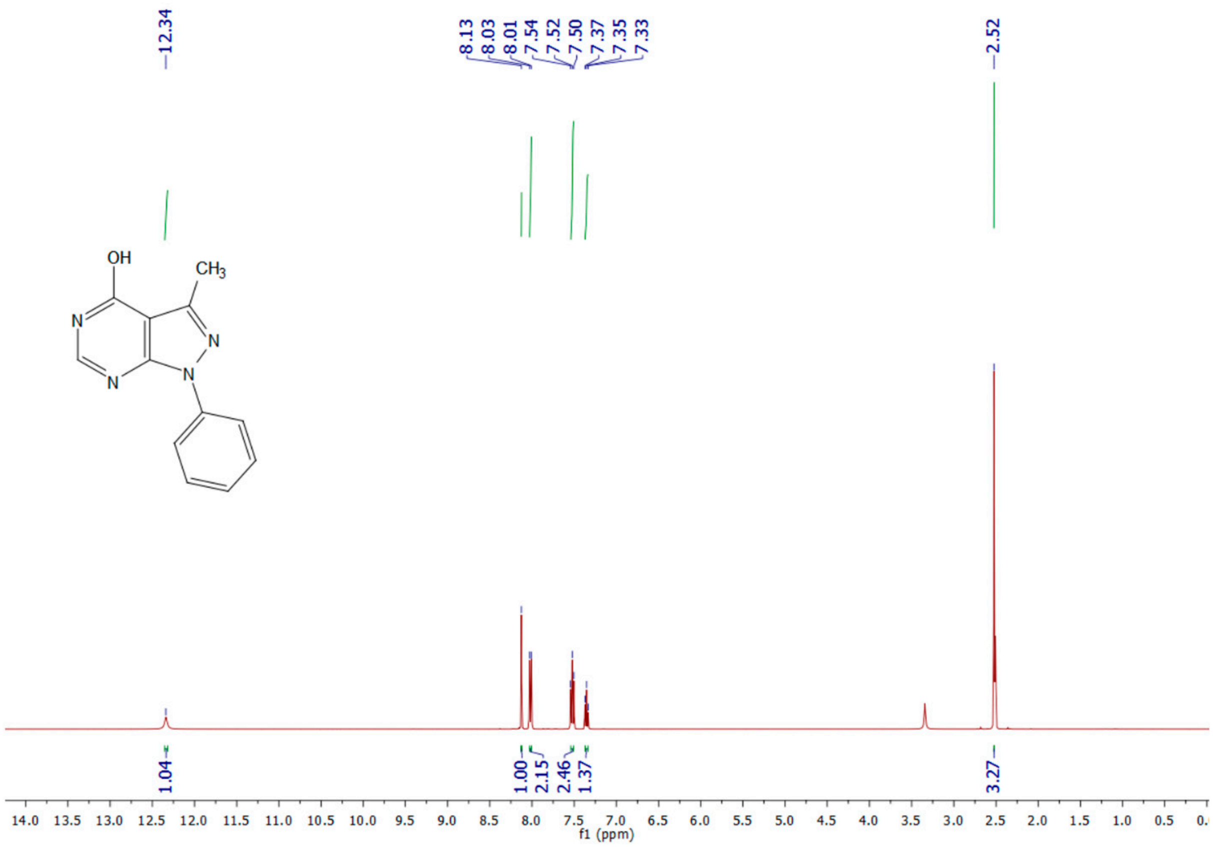

molecules

MDPI

**Figure S6**
$^{13}$C NMR spectrum of compound **P1**.

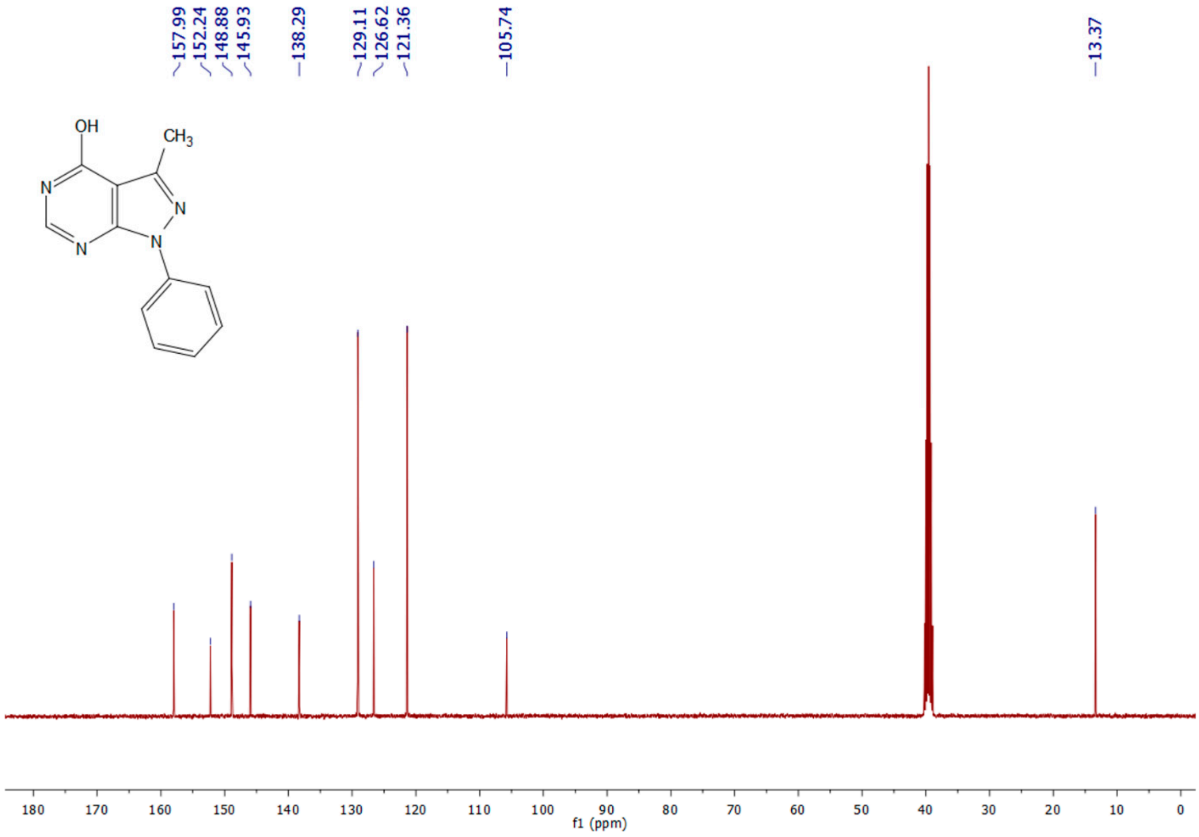

molecules MDPI

**Figure S7**

$^{1}$H NMR spectrum of compound **P2**.

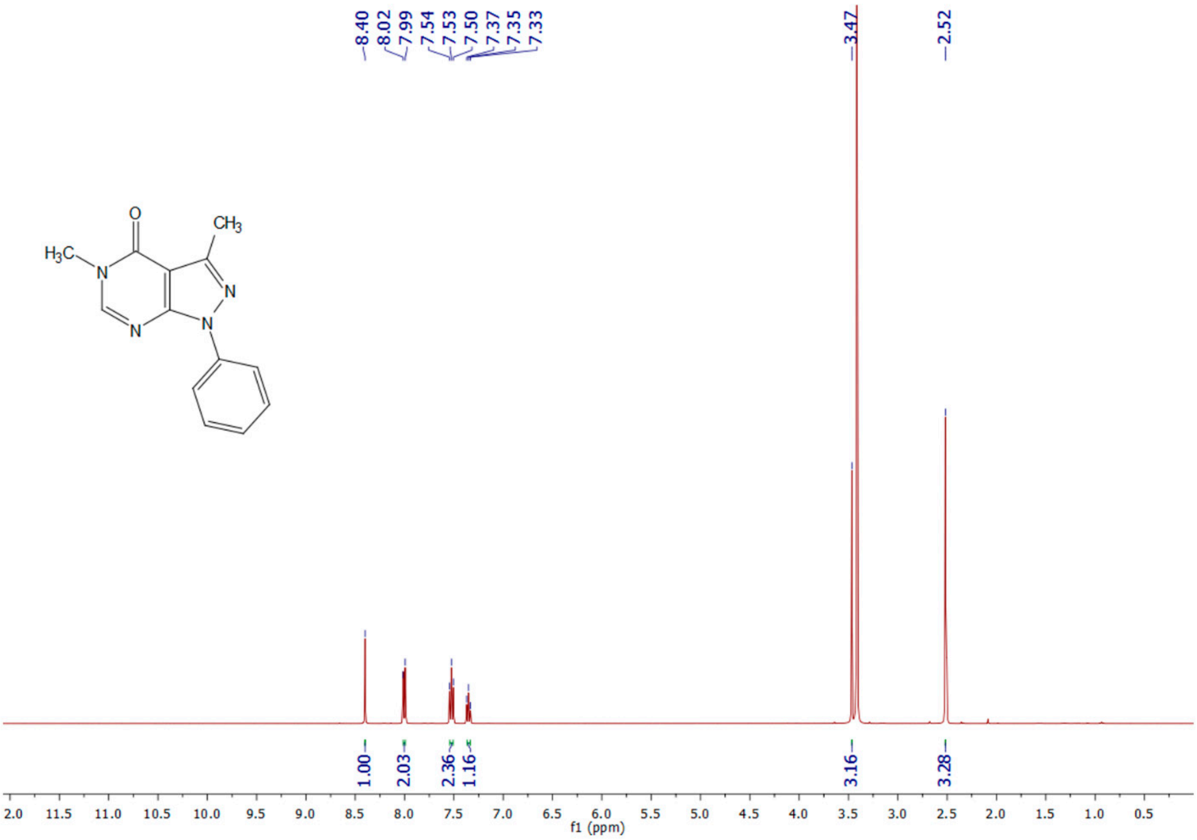

molecules MDPI

**Figure S8**
$^{13}$C NMR spectrum of compound P**2**.

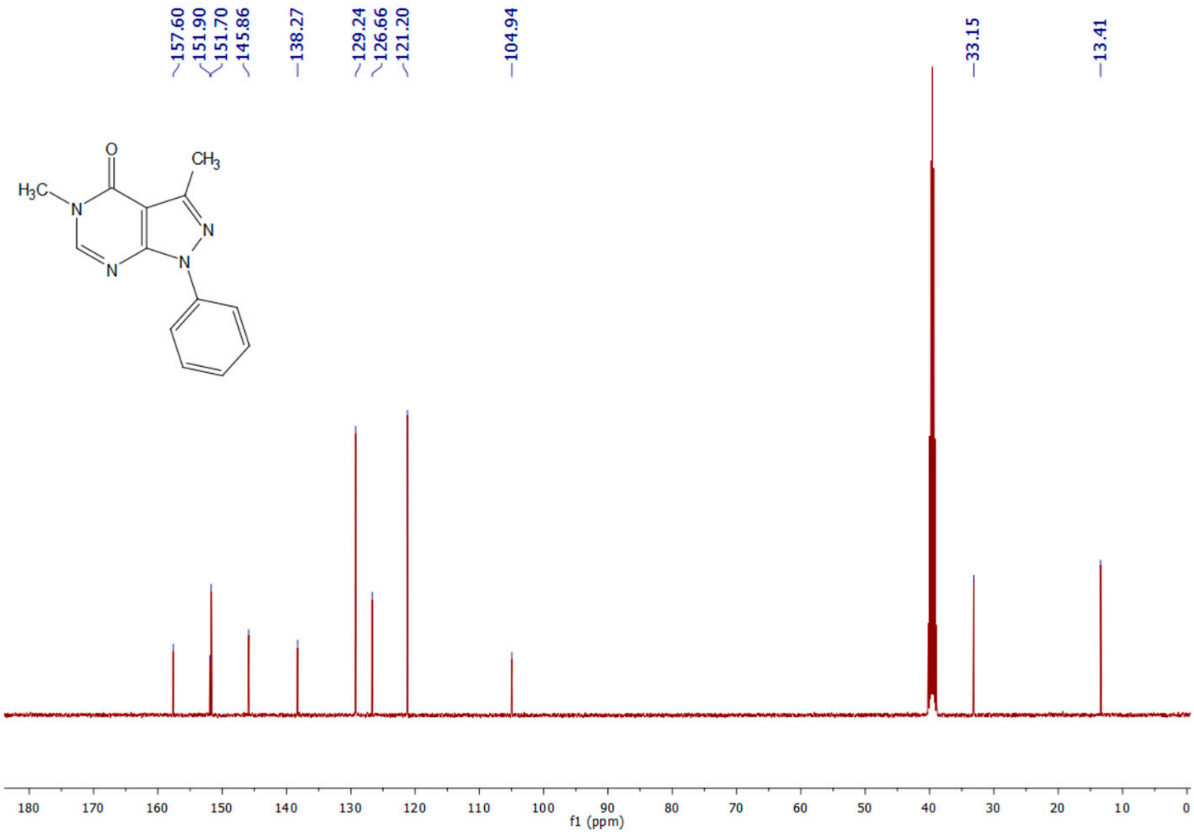

molecules

MDPI

**Figure S9**

$^{1}$H NMR spectrum of compound **P3**.

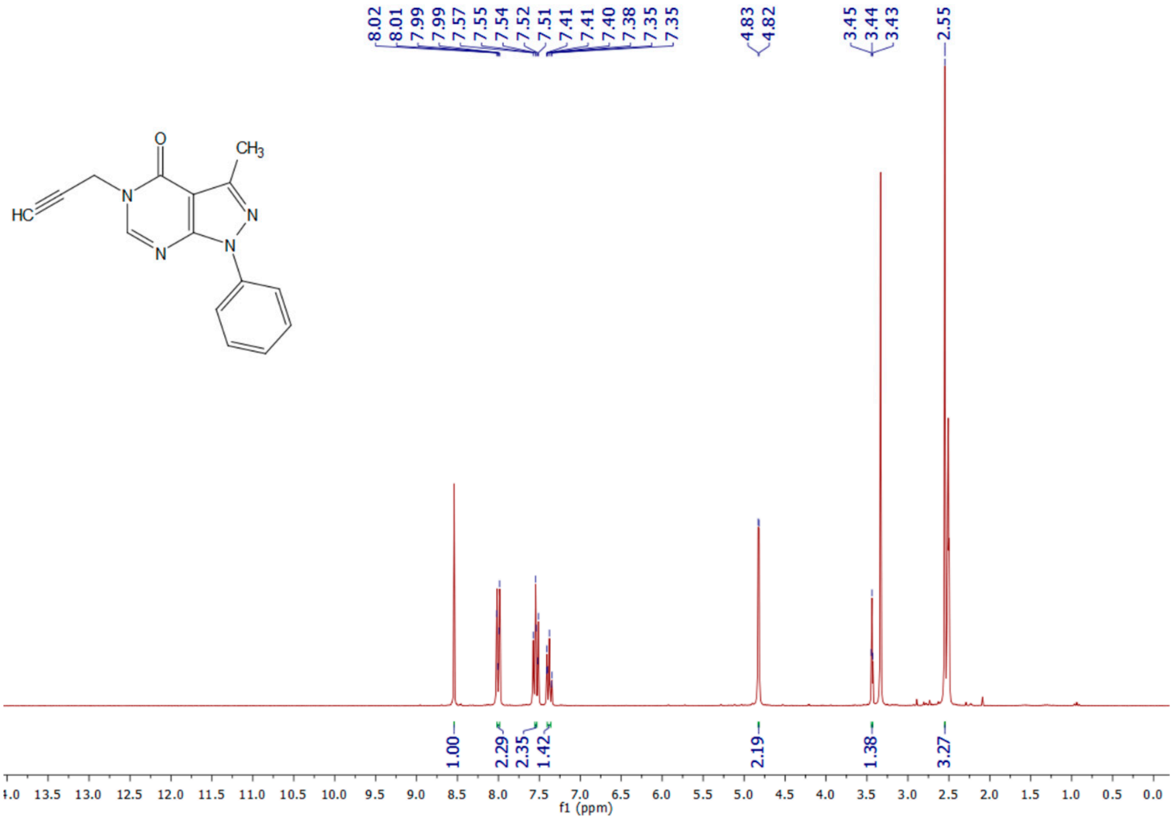

molecules MDPI

**Figure S10**
$^{13}$C NMR spectrum of compound **P3**.

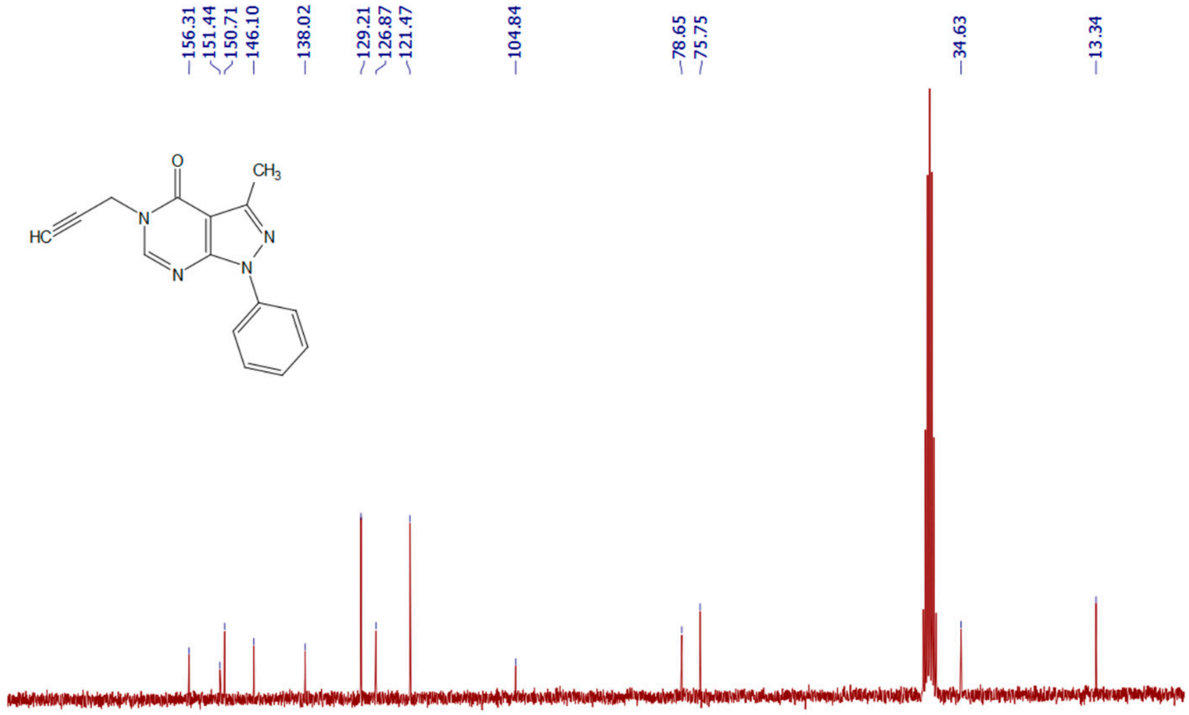

molecules

MDPI

**Figure S11**
$^{1}$H NMR spectrum of compound **P4**.

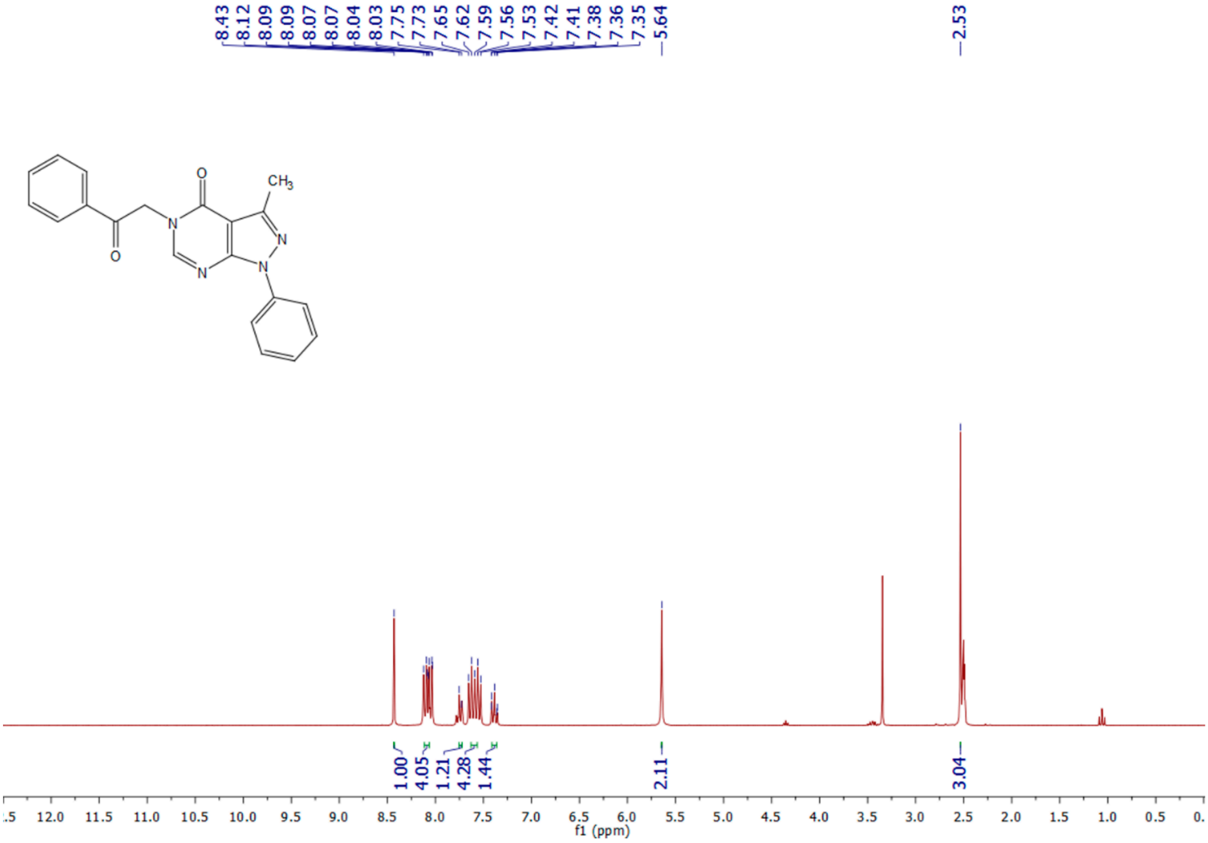

molecules MDPI

**Figure S12**
$^{13}C$ NMR spectrum of compound **P4**.

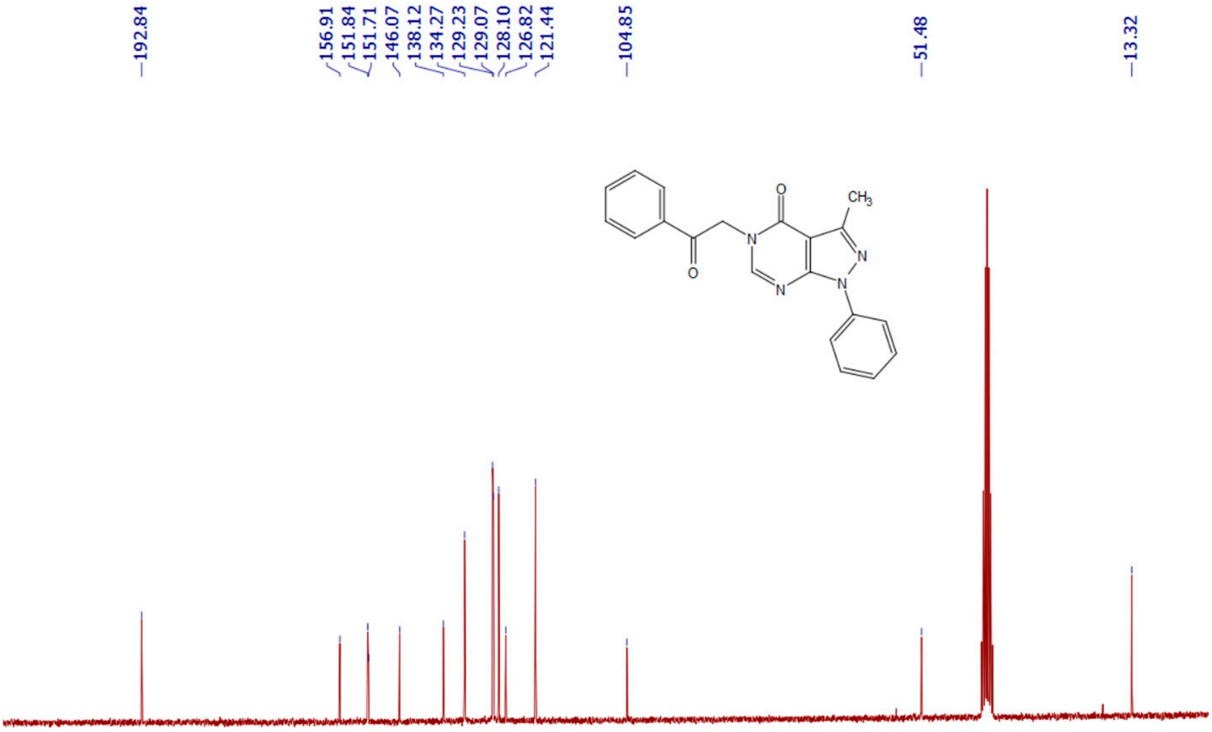

molecules
MDPI

**Figure S13**

Correlation curves between experimental and calculated bond lengths, bond angles, and dihedral angles of **P1-P4**.

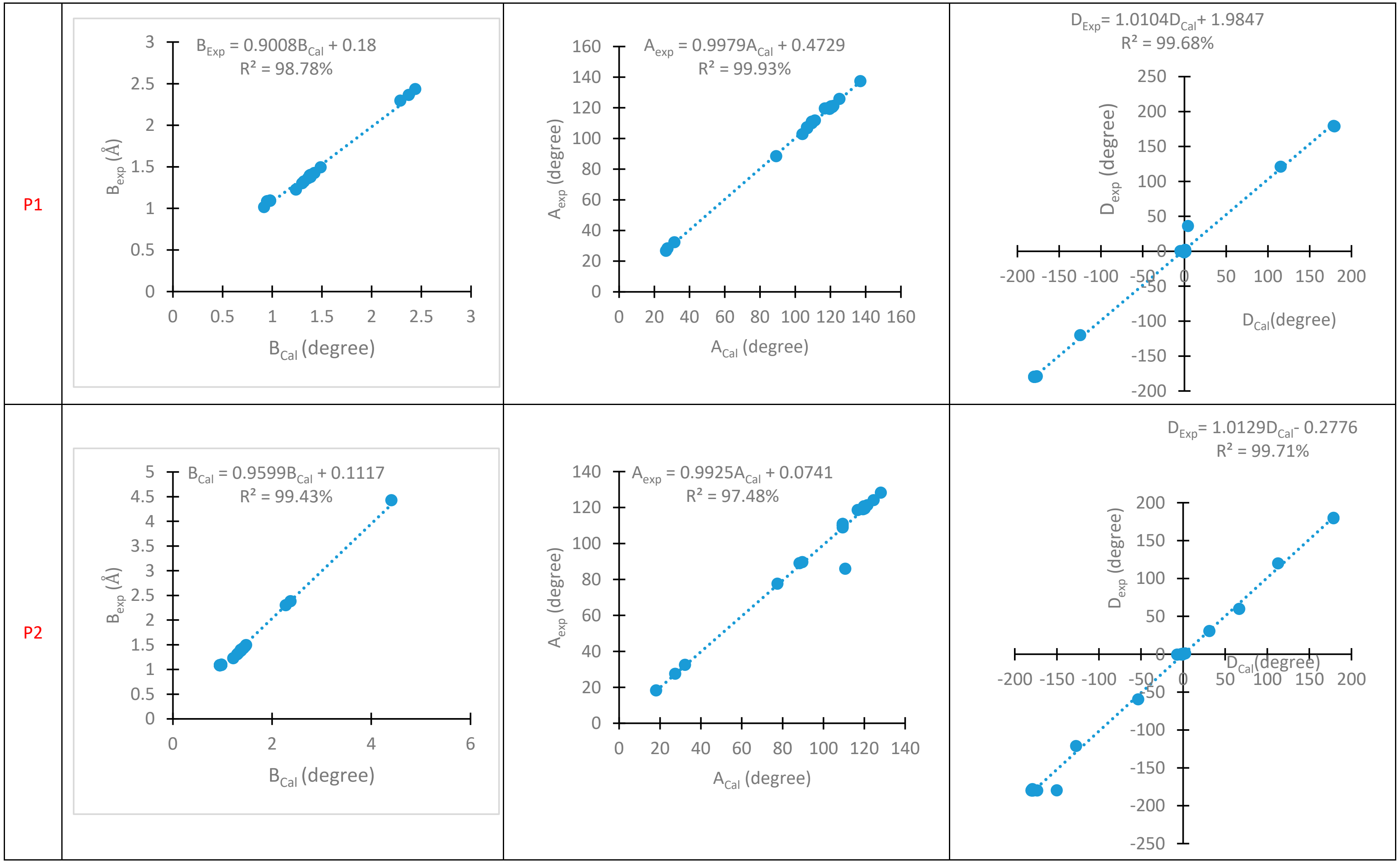

P3

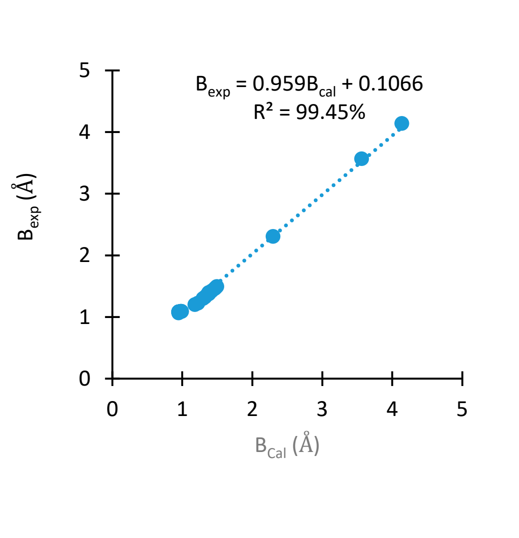


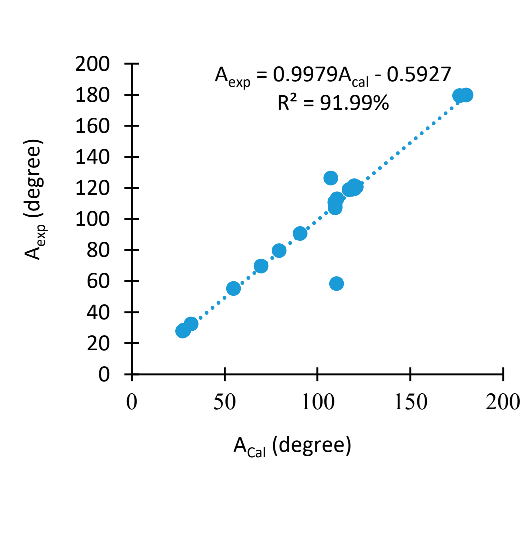


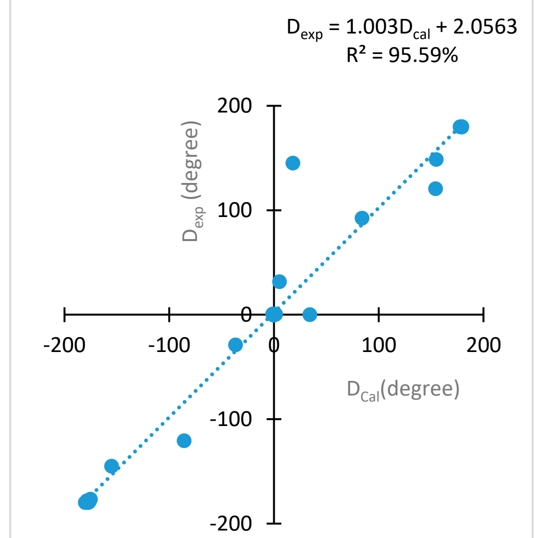


P4

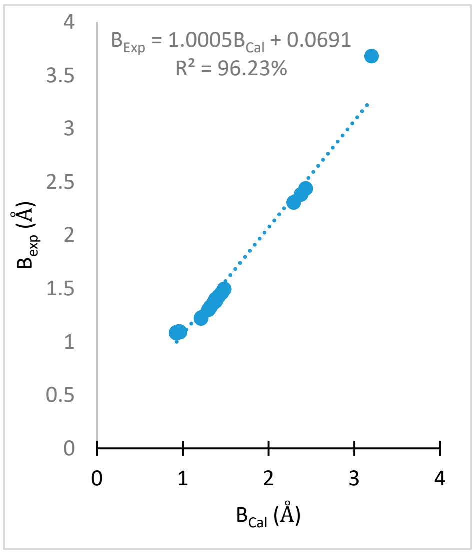


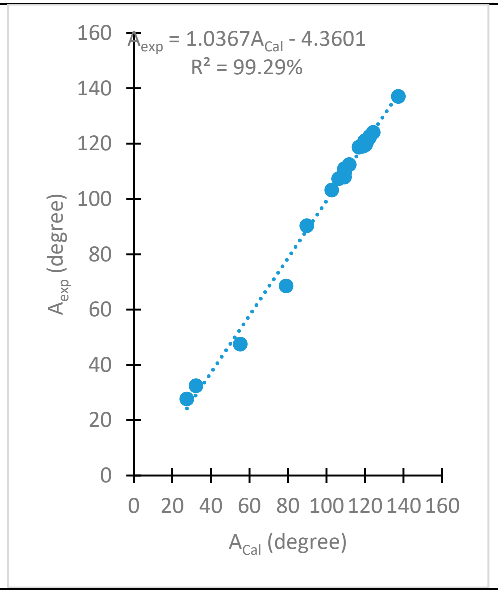


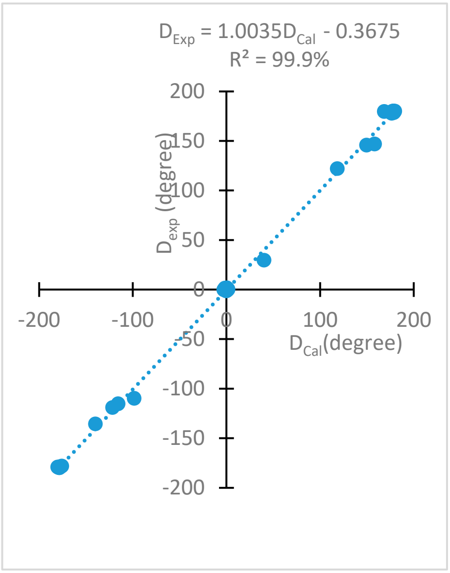


Note: B for bond length, A for bond angle, and D for dihedral angle

molecules

**Table S1**
The crystal data and structure refinement details of **P1**.

| | |
|---|---|
| Chemical formula | $C_{12}H_{10}N_4O$ |
| Formula weight | 226.24 g/mol |
| Temperature | 171(2) K |
| Wavelength | 0.71073 Å |
| Crystal size | 0.069 x 0.095 x 0.289 mm |
| Crystal habit | colorless column |
| Crystal system, Space group | monoclinic, P 1 21/n 1 |
| Cell parameters | *a* = 5.0757(2) Å |
| | *b* = 12.2023(6) Å |
| | *c* = 16.9251(8) Å |
| | α = 90° (2)°<br>*β* = 95.009(2)°<br>γ = 90° (3)° |
| Volume | 1044.26(8) $Å^3$ |
| *Z* | 4 |
| Density (calculated) | 1.439 $g/cm^3$ |
| Absorption coefficient | 0.098 $mm^{-1}$ |
| $F_{000}$ | 472 |
| Index ranges | -7<=h<=7 |
| | -17<=k<=17 |
| | -23<=l<=23 |
| Reflections collected | 41497 |
| Independent reflections | 3071 [R(int) = 0.0554] |
| Absorption correction | Numerical |
| Refinement method | Full-matrix least-squares on $F^2$ |
| Data/restraints/parameters | 3071 / 1 / 159 |
| Goodness-of-fit on $F^2$ | 1.033 |
| Final [$I > 2\sigma(I)$] | R1 = 0.0432, $wR_2$ = 0.1047 |
| *R* indices (all data) | R1 = 0.0665, $wR_2$ = 0.1185 |
| Largest diff. Peak and hole | 0.233 and -0.226 $eÅ^{-3}$ |

molecules

MDPI

**Table S2**

The crystal data and structure refinement details of compound **P2**.

| | |
|---|---|
| Chemical formula | $C_{13}H_{12}N_4O$ |
| Formula weight | 240.27 g/mol |
| Temperature | 150(2) K |
| Wavelength | 0.71073 Å |
| Crystal size | 0.118 x 0.263 x 0.328 mm |
| Crystal habit | colorless plate |
| Crystal system, Space group | Monoclinic, P 1 21/c 1 |
| Cell parameters | *a* = 10.551(4) Å |
| | *b* = 14.833(6) Å |
| | *c* = 7.593(3) Å |
| | α = 90°<br>*β* = 101.398(14)°<br>γ = 90° |
| Volume | 1164.9(8) $Å^3$ |
| *Z* | 4 |
| Density (calculated) | 1.370 $g/cm^3$ |
| Absorption coefficient | 0.092 $mm^{-1}$ |
| $F_{000}$ | 504 |
| Index ranges | -17<=h<=17 |
| | -24<=k<=24 |
| | -12<=l<=12 |
| Reflections collected | 70045 |
| Independent reflections | 5714 [R(int) = 0.0715] |
| Absorption correction | Multi-scan |
| Refinement method | Full-matrix least-squares on $F^2$ |
| Data/restraints/parameters | 5714 / 0 / 165 |
| Goodness-of-fit on $F^2$ | 1.047 |
| Final [$I > 2\sigma(I)$] | R1 = 0.0573, $wR_2$ = 0.1465 |
| *R* indices (all data) | R1 = 0.1097, $wR_2$ = 0.1700 |
| Largest diff. Peak and hole | 0.288 and -0.176 $eÅ^{-3}$ |

molecules

MDPI

**Table S3**

The crystal data and structure refinement details of compound **P3**.

| | |
|---|---|
| Chemical formula | $C_{15}H_{12}N_4O$ |
| Formula weight | 264.29 g/mol |
| Temperature | 150(2) K |
| Wavelength | 0.71073 Å |
| Crystal size | 0.252 x 0.295 x 0.342 mm |
| Crystal system, Space group | Monoclinic, P 1 21/c 1 |
| Cell parameters | *a* = 7.2768(4) Å |
| | *b* = 16.2369(8) Å |
| | *c* = 10.7931(5) Å |
| | α = 90°<br>*β* = 91.482(2)°<br>γ = 90° |
| Volume | 1274.81(11) $Å^3$ |
| *Z* | 4 |
| Density (calculated) | 1.377 $g/cm^3$ |
| Absorption coefficient | 0.091 $mm^{-1}$ |
| $F_{000}$ | 552 |
| Index ranges | -9<=h<=9 |
| | -21<=k<=21 |
| | -14<=l<=14 |
| Reflections collected | 53166 |
| Independent reflections | 3199 [R(int) = 0.0412] |
| Absorption correction | Numerical mu Calculated |
| Refinement method | Full-matrix least-squares on $F^2$ |
| Data/restraints/parameters | 3199 / 0 / 182 |
| Goodness-of-fit on $F^2$ | 1.044 |
| Final [$I > 2\sigma(I)$] | R1 = 0.0433, $wR_2$ = 0.1101 |
| *R* indices (all data) | R1 = 0.0520, $wR_2$ = 0.1162 |
| Largest diff. Peak and hole | 0.446 and -0.199 $eÅ^{-3}$ |

molecules MDPI

**Table S4**
The crystal data and structure refinement details of compound **P4**.

| | |
|---|---|
| Chemical formula | $C_{20}H_{16}N_4O_2$ |
| Formula weight | 344.37 g/mol |
| Temperature | 272(2) K |
| Wavelength | 0.71073 Å |
| Crystal size | 0.126 x 0.384 x 0.452 mm |
| Crystal habit | colorless plate |
| Crystal system, Space group | Monoclinic, P 1 21/c 1 |
| Cell parameters | *a* = 13.2177(7) Å |
| | *b* = 14.9602(9) Å |
| | *c* = 8.5784(5) Å |
| | α = 90°<br>*β* = 105.325(2)°<br>γ = 90° |
| Volume | 1635.97(16) $Å^3$ |
| *Z* | 4 |
| Density (calculated) | 1.398 $g/cm^3$ |
| Absorption coefficient | 0.094 $mm^{-1}$ |
| $F_{000}$ | 720 |
| Index ranges | -19<=h<=20 |
| | -23<=k<=23 |
| | -13<=l<=13 |
| Reflections collected | 85204 |
| Independent reflections | 6209 [R(int) = 0.0402] |
| Absorption correction | Numerical mu Calculated |
| Refinement method | Full-matrix least-squares on $F^2$ |
| Data/restraints/parameters | 6209 / 0 / 236 |
| Goodness-of-fit on $F^2$ | 1.051 |
| Final [$I > 2\sigma(I)$] | R1 = 0.0439, $wR_2$ = 0.1183 |
| *R* indices (all data) | R1 = 0.0546, $wR_2$ = 0.1298 |
| Largest diff. Peak and hole | 0.420 and -0.294 $eÅ^{-3}$ |

molecules

MDPI

**The coordinate and energy information of P1-P4 after optimization**

**P1; Energy: -757.76 au**

**Careteisan coordinate:**

| Center Number | Atomic Number | Atomic Type | Coordinates (Angstroms) X | Y | Z |
|---|---|---|---|---|---|
| 1 | 8 | 0 | 6.323995 | 4.625521 | 9.042154 |
| 2 | 7 | 0 | 5.305075 | 5.810941 | 7.363178 |
| 3 | 1 | 0 | 5.908924 | 6.593208 | 7.586003 |
| 4 | 7 | 0 | 3.567456 | 5.020614 | 5.973027 |
| 5 | 7 | 0 | 2.952357 | 2.729521 | 6.516243 |
| 6 | 7 | 0 | 3.319418 | 1.773851 | 7.435572 |
| 7 | 6 | 0 | 5.482316 | 4.647401 | 8.153540 |
| 8 | 6 | 0 | 4.393648 | 5.947183 | 6.358738 |
| 9 | 1 | 0 | 4.381901 | 6.913108 | 5.865824 |
| 10 | 6 | 0 | 3.688397 | 3.862779 | 6.682171 |
| 11 | 6 | 0 | 4.288105 | 2.290887 | 8.173082 |
| 12 | 6 | 0 | 4.570786 | 3.617563 | 7.741949 |
| 13 | 6 | 0 | 1.898603 | 2.458104 | 5.596848 |
| 14 | 6 | 0 | 1.935109 | 2.982082 | 4.302788 |
| 15 | 1 | 0 | 2.763189 | 3.602994 | 3.992012 |
| 16 | 6 | 0 | 0.893791 | 2.698744 | 3.421589 |
| 17 | 1 | 0 | 0.922913 | 3.107017 | 2.417897 |
| 18 | 6 | 0 | -0.168930 | 1.887776 | 3.817058 |
| 19 | 1 | 0 | -0.972798 | 1.666584 | 3.124635 |
| 20 | 6 | 0 | -0.189304 | 1.359534 | 5.107975 |
| 21 | 1 | 0 | -1.010791 | 0.727346 | 5.424855 |
| 22 | 6 | 0 | 0.837821 | 1.644702 | 6.003136 |
| 23 | 1 | 0 | 0.829077 | 1.241958 | 7.006969 |
| 24 | 6 | 0 | 4.929483 | 1.506915 | 9.270103 |
| 25 | 1 | 0 | 4.481765 | 0.514614 | 9.329539 |
| 26 | 1 | 0 | 4.805031 | 2.010749 | 10.232009 |
| 27 | 1 | 0 | 6.003463 | 1.402803 | 9.097508 |

molecules

MDPI

**P2; Energy: -797.07 au**

**Careteisan coordinate:**

| Center Number | Atomic Number | Atomic Type | Coordinates (Angstroms) X | Y | Z |
|---|---|---|---|---|---|
| 1 | 8 | 0 | 7.464429 | 5.506600 | 7.678859 |
| 2 | 7 | 0 | 6.248835 | 7.368683 | 7.091457 |
| 3 | 7 | 0 | 4.027250 | 3.750958 | 5.516168 |
| 4 | 7 | 0 | 3.547791 | 5.022373 | 5.294799 |
| 5 | 7 | 0 | 4.210244 | 7.297297 | 5.864688 |
| 6 | 6 | 0 | 6.473521 | 5.959969 | 7.118696 |
| 7 | 6 | 0 | 5.432075 | 5.245085 | 6.440882 |
| 8 | 6 | 0 | 5.154387 | 3.869621 | 6.197510 |
| 9 | 6 | 0 | 4.378077 | 5.948829 | 5.852415 |
| 10 | 6 | 0 | 5.160743 | 7.931774 | 6.488639 |
| 11 | 1 | 0 | 5.108313 | 9.013264 | 6.550864 |
| 12 | 6 | 0 | 7.247298 | 8.216094 | 7.754992 |
| 13 | 1 | 0 | 7.316496 | 7.951746 | 8.809966 |
| 14 | 1 | 0 | 6.946811 | 9.257164 | 7.656114 |
| 15 | 1 | 0 | 8.223607 | 8.069967 | 7.293345 |
| 16 | 6 | 0 | 5.948951 | 2.670053 | 6.596470 |
| 17 | 1 | 0 | 5.451373 | 1.762292 | 6.253800 |
| 18 | 1 | 0 | 6.065164 | 2.624512 | 7.682342 |
| 19 | 1 | 0 | 6.953292 | 2.708638 | 6.167086 |
| 20 | 6 | 0 | 2.315262 | 5.202831 | 4.605546 |
| 21 | 6 | 0 | 1.323649 | 4.224979 | 4.722636 |
| 22 | 1 | 0 | 1.504553 | 3.354249 | 5.337938 |
| 23 | 6 | 0 | 0.120363 | 4.382027 | 4.040799 |
| 24 | 1 | 0 | -0.645762 | 3.620728 | 4.132753 |
| 25 | 6 | 0 | -0.104034 | 5.510661 | 3.252357 |
| 26 | 1 | 0 | -1.043930 | 5.630869 | 2.726371 |
| 27 | 6 | 0 | 0.890628 | 6.481250 | 3.142730 |
| 28 | 1 | 0 | 0.729558 | 7.358303 | 2.526443 |
| 29 | 6 | 0 | 2.104943 | 6.331539 | 3.809282 |
| 30 | 1 | 0 | 2.877612 | 7.081144 | 3.716155 |

molecules

MDPI

**P3; Energy: -873.23 au**

**Careteisan coordinate:**

| Center Number | Atomic Number | Atomic Type | Coordinates (Angstroms) X | Y | Z |
|---|---|---|---|---|---|
| 1 | 8 | 0 | 4.131853 | 5.391265 | 1.288279 |
| 2 | 7 | 0 | 4.669722 | 6.678315 | 5.184812 |
| 3 | 7 | 0 | 3.947050 | 5.090705 | 3.568196 |
| 4 | 7 | 0 | 5.834446 | 9.145813 | 2.886243 |
| 5 | 7 | 0 | 5.621541 | 8.672378 | 4.160759 |
| 6 | 6 | 0 | 5.052860 | 7.435767 | 4.121504 |
| 7 | 6 | 0 | 4.137826 | 5.542541 | 4.845638 |
| 8 | 1 | 0 | 3.801327 | 4.869634 | 5.627271 |
| 9 | 6 | 0 | 4.324625 | 5.841442 | 2.409337 |
| 10 | 6 | 0 | 4.906505 | 7.100065 | 2.772913 |
| 11 | 6 | 0 | 5.414432 | 8.214526 | 2.046172 |
| 12 | 6 | 0 | 3.343752 | 3.759291 | 3.358317 |
| 13 | 1 | 0 | 2.599910 | 3.593545 | 4.140201 |
| 14 | 1 | 0 | 2.824511 | 3.791579 | 2.400330 |
| 15 | 6 | 0 | 4.330689 | 2.680889 | 3.356957 |
| 16 | 6 | 0 | 5.132639 | 1.785870 | 3.362012 |
| 17 | 1 | 0 | 5.845161 | 0.995521 | 3.361389 |
| 18 | 6 | 0 | 5.513580 | 8.402273 | 0.568301 |
| 19 | 1 | 0 | 5.959056 | 9.372215 | 0.345557 |
| 20 | 1 | 0 | 4.526324 | 8.348813 | 0.102310 |
| 21 | 1 | 0 | 6.125683 | 7.617549 | 0.116815 |
| 22 | 6 | 0 | 5.947721 | 9.487355 | 5.282341 |
| 23 | 6 | 0 | 5.834772 | 10.875157 | 5.166190 |
| 24 | 1 | 0 | 5.497301 | 11.305670 | 4.233217 |
| 25 | 6 | 0 | 6.163590 | 11.683174 | 6.250831 |
| 26 | 1 | 0 | 6.076271 | 12.759553 | 6.157673 |
| 27 | 6 | 0 | 6.593659 | 11.115999 | 7.450509 |
| 28 | 1 | 0 | 6.844563 | 11.748690 | 8.293868 |
| 29 | 6 | 0 | 6.702276 | 9.730227 | 7.556513 |
| 30 | 1 | 0 | 7.043313 | 9.280134 | 8.481698 |
| 31 | 6 | 0 | 6.388813 | 8.909534 | 6.475154 |
| 32 | 1 | 0 | 6.482328 | 7.836013 | 6.556290 |

molecules

MDPI

**P4; Energy: -1141.54 au**

**Careteisan coordinate:**

| Center Number | Atomic Number | Atomic Type | Coordinates (Angstroms) X | Y | Z |
|---|---|---|---|---|---|
| 1 | 8 | 0 | 2.401383 | 4.733593 | 3.505132 |
| 2 | 8 | 0 | 1.925814 | 7.600187 | 1.249403 |
| 3 | 7 | 0 | 3.591854 | 6.681797 | 3.186180 |
| 4 | 7 | 0 | 5.822838 | 6.759508 | 2.363167 |
| 5 | 7 | 0 | 6.733820 | 4.539478 | 1.947361 |
| 6 | 7 | 0 | 6.309683 | 3.236515 | 2.077880 |
| 7 | 6 | 0 | 3.451759 | 5.258748 | 3.157887 |
| 8 | 6 | 0 | 4.731045 | 7.323955 | 2.785186 |
| 9 | 1 | 0 | 4.695007 | 8.406419 | 2.846665 |
| 10 | 6 | 0 | 5.750712 | 5.401891 | 2.327940 |
| 11 | 6 | 0 | 4.653503 | 4.623417 | 2.705437 |
| 12 | 6 | 0 | 5.065844 | 3.272047 | 2.525906 |
| 13 | 6 | 0 | 4.292901 | 2.016976 | 2.764536 |
| 14 | 1 | 0 | 3.398760 | 1.986185 | 2.136739 |
| 15 | 1 | 0 | 3.959423 | 1.956378 | 3.803547 |
| 16 | 1 | 0 | 4.913703 | 1.149308 | 2.539252 |
| 17 | 6 | 0 | 8.061287 | 4.811345 | 1.509826 |
| 18 | 6 | 0 | 9.082533 | 3.920092 | 1.849178 |
| 19 | 1 | 0 | 8.850889 | 3.044768 | 2.440754 |
| 20 | 6 | 0 | 10.381721 | 4.168310 | 1.415483 |
| 21 | 1 | 0 | 11.171856 | 3.474517 | 1.678745 |
| 22 | 6 | 0 | 10.669131 | 5.302543 | 0.655986 |
| 23 | 1 | 0 | 11.682840 | 5.494059 | 0.323881 |
| 24 | 6 | 0 | 9.643291 | 6.185900 | 0.323037 |
| 25 | 1 | 0 | 9.854689 | 7.065360 | -0.274248 |
| 26 | 6 | 0 | 8.335915 | 5.943526 | 0.739171 |
| 27 | 1 | 0 | 7.539776 | 6.623540 | 0.471585 |
| 28 | 6 | 0 | 2.428664 | 7.465138 | 3.582201 |
| 29 | 1 | 0 | 2.760813 | 8.369030 | 4.097766 |
| 30 | 1 | 0 | 1.848196 | 6.868788 | 4.284904 |
| 31 | 6 | 0 | 1.553434 | 7.853845 | 2.378676 |
| 32 | 6 | 0 | 0.269222 | 8.560234 | 2.651041 |
| 33 | 6 | 0 | -0.172125 | 8.849393 | 3.950856 |
| 34 | 1 | 0 | 0.412158 | 8.558697 | 4.814926 |
| 35 | 6 | 0 | -1.378161 | 9.516409 | 4.150657 |
| 36 | 1 | 0 | -1.712302 | 9.734839 | 5.158043 |
| 37 | 6 | 0 | -2.153916 | 9.898811 | 3.057210 |
| 38 | 1 | 0 | -3.093298 | 10.416408 | 3.214841 |
| 39 | 6 | 0 | -1.722616 | 9.614205 | 1.759423 |
| 40 | 1 | 0 | -2.326198 | 9.910553 | 0.909465 |
| 41 | 6 | 0 | -0.519467 | 8.950320 | 1.557536 |
| 42 | 1 | 0 | -0.172384 | 8.722967 | 0.557438 |
